# Two β-glucuronosyltransferases involved in the biosynthesis of type II arabinogalactans function in mucilage polysaccharide matrix organization in *Arabidopsis thaliana*

**DOI:** 10.1186/s12870-021-03012-7

**Published:** 2021-05-29

**Authors:** Oyeyemi O. Ajayi, Michael A. Held, Allan M. Showalter

**Affiliations:** 1 Department of Environmental and Plant Biology, Ohio University, Athens, OH 45701 USA; 2 Molecular and Cellular Biology Program, Ohio University, Athens, OH 45701 USA; 3 Department of Chemistry and Biochemistry, Ohio University, Athens, OH 45701 USA

**Keywords:** Arabinogalactan-protein, Glucuronosyltransferases, Glucuronic acid, Mucilage, Seed, Mutant, Arabidopsis, Sugar, Genetics

## Abstract

**Background:**

Arabinogalactan-proteins (AGPs) are heavily glycosylated with type II arabinogalactan (AG) polysaccharides attached to hydroxyproline residues in their protein backbone. Type II AGs are necessary for plant growth and critically important for the establishment of normal cellular functions. Despite the importance of type II AGs in plant development, our understanding of the underlying role of these glycans/sugar residues in mucilage formation and seed coat epidermal cell development is poorly understood and far from complete. One such sugar residue is the glucuronic acid residues of AGPs that are transferred onto AGP glycans by the action of β-glucuronosyltransferase genes/enzymes.

**Results:**

Here, we have characterized two β-glucuronosyltransferase genes, *GLCAT14A* and *GLCAT14C*, that are involved in the transfer of β-glucuronic acid (GlcA) to type II AGs. Using a reverse genetics approach, we observed that *glcat14a-1* mutants displayed subtle alterations in mucilage pectin homogalacturonan (HG) compared to wild type (WT), while *glcat14a-1glcat14c-1* mutants displayed much more severe mucilage phenotypes, including loss of adherent mucilage and significant alterations in cellulose ray formation and seed coat morphology. Monosaccharide composition analysis showed significant alterations in the sugar amounts of *glcat14a-1glcat14c-1* mutants relative to WT in the adherent and non-adherent seed mucilage. Also, a reduction in total mucilage content was observed in *glcat14a-1glcat14c-1* mutants relative to WT. In addition, *glcat14a-1glcat14c-1* mutants showed defects in pectin formation, calcium content and the degree of pectin methyl-esterification (DM) as well as reductions in crystalline cellulose content and seed size.

**Conclusions:**

These results raise important questions regarding cell wall polymer interactions and organization during mucilage formation. We propose that the enzymatic activities of GLCAT14A and GLCAT14C play partially redundant roles and are required for the organization of the mucilage matrix and seed size in *Arabidopsis thaliana*. This work brings us a step closer towards identifying potential gene targets for engineering plant cell walls for industrial applications.

**Supplementary Information:**

The online version contains supplementary material available at 10.1186/s12870-021-03012-7.

## Background

Normal plant development depends critically on the interactions between different components of the plant cell wall. This dynamic structure defines the plant morphological architecture and is responsible for cell shape, cell adhesion and organ cohesion [1]. Plant cell walls are initiated by the synthesis, secretion, modification and crosslinking of individual wall components– cellulose, hemicellulose, pectin and hydroxyproline-rich glycoproteins- and are synthesized by the coordinated action of a myriad of glycosyltransferases. Understanding the underlying mechanisms involved in the assembly of a complex polysaccharide network and elucidating their biological roles is not a trivial task [2], and remains to date a key goal for scientists interested in the manipulation of plant cell wall structure to better understand its physiological functions and allow for its commercial exploitation.

One model system that is gaining increasing recognition and significance for the study of cell wall polysaccharide interactions is the Arabidopsis seed coat epidermis (SCE), also referred to as Mucilage Secretary Cells (MSC) [1]. The SCE is an excellent model system for understanding the genetic basis of cell wall biosynthesis, secretion, assembly and modification [3, 4] because large amounts of cell wall polysaccharides can be extracted with ease and analyzed in a short timeframe. Between 5- and 8-days post anthesis (DPA), large amounts of pectins are secreted to the apoplastic space at the junction of the outer tangential and radial primary walls, forming a donut-shaped pocket of mucilage around a cytoplasmic column [4]. The epidermal cells then synthesize a volcano-shaped secondary wall (9 to 11 DPA) called the columella, which protrudes through the center of the mucilage pocket and connects to the primary wall. When dry, mature seeds imbibe water, rapid mucilage expansion ruptures the tangential SCE to release the polysaccharide-rich mucilage that is organized in two distinct layers: an outer, water soluble non-adherent layer and an inner, adherent layer that remains tightly attached to the seed coat surface. Arabidopsis mucilage is composed primarily of unbranched Rhamnogalacturonan-1 (RG-I), with small quantities of Homogalacturonan (HG), cellulose, and arabinoxylan found in the inner layer [3, 5]. Several attempts have been made to better understand the functional roles of the glycosyltransferases involved in cell wall biosynthesis, secretion, and delivery of the mucilage polymers through the analysis of mucilage mutants. In recent years, genetic mutants that lack functional enzymes required for mucilage biosynthesis and extrusion have been identified and characterized, but many others await functional investigation.

Work to date has identified several genes/proteins involved in mucilage biosynthesis, including a fasciclin-like arabinogalactan-protein (AGP) named SALT OVERLY SENSITIVE 5 (SOS5), GALT2 and GALT5, two galactosyltransferases responsible for initiating glycosylation of AGPs, and a receptor-like kinase called FEI2; individual as well as higher order mutants corresponding to these genes/proteins are characterized by mucilage pectin repartitioning and the marked absence of cellulosic rays, while the diffuse cellulose staining remains intact [6]. As SOS5, which is also known as FLA4, is the only well characterized AGP reported to be involved in mucilage biosynthesis, the contribution of the SOS5 glycan moieties and potentially other AGPs to mucilage formation is far from complete and presents an enigma worth unraveling.

AGPs are a family of hydroxyproline-rich glycoproteins that are extensively glycosylated with type II AGs that are covalently attached to hydroxyproline residues in the AGP protein backbone [7, 8]. An individual type II AG glycan consists of a β-1,3-galactan backbone with β -1,6-galactosyl branches that are decorated with arabinosyl residues and often with other minor sugar residues, such as glucuronic acid (GlcA), rhamnose (Rha), and Fuc [8, 9]. Although their exact roles in mucilage formation are still unclear, the interaction of AGPs with wall polysaccharides, their involvement in intracellular signaling cascades, and their influence on a wide variety of biological processes are known [8, 10]. Notably, the complexity of the cell wall polymer network with respect to AGPs is perhaps best illustrated by the finding that AGPs form covalent linkages to both RG- I and arabinoxylan [10].

Three glucuronosyltransferases (GLCATs), GLCAT14A, GLCAT14B and GLCAT14C were functionally characterized and found to transfer GlcA residues to AGPs [11], while two additional GLCATs (GLCAT14D and GLCAT14E) were also reported to be involved in the glucuronidation of AGPs [12]. Here, we present evidence that two GLCATs (GLCAT14A and GLCAT14C) belonging to family GT14 in the Carbohydrate-Active Enzymes (CAZy) classification system (http://www.cazy.org; [13]) are critically important in mucilage matrix formation in Arabidopsis.

## Results

### Phylogenetic, mutant characterization and gene expression analyses of the *GLCAT14A* and *GLCAT14C* genes

**Fig. 1:**
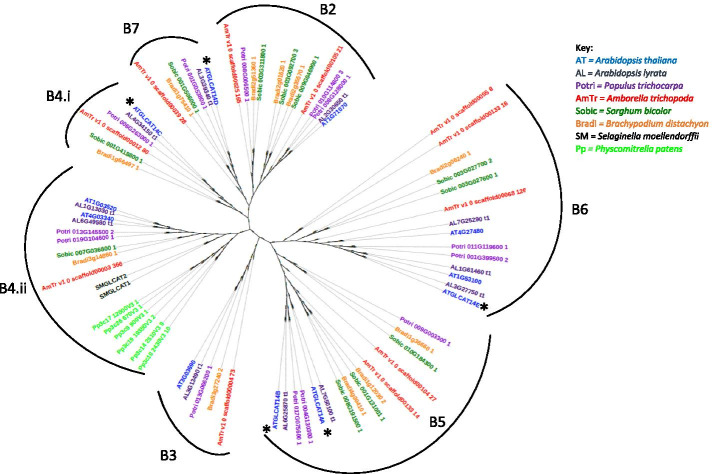
Phylogenetic tree of CAZy GT14 proteins from eight species. Five GT14s (denoted by an asterisk) have previously shown GlcA transferase (GlcAT) activity. The species protein sequences used in this phylogenetic study are indicated in the figure legend. The clades were labelled as in previous reports [16, 17]. The GLCAT protein sequences of the investigated species were manually extracted from the Phytozome database version v12.1.6 (https://phytozome.jgi.doe.gov/pz/portal.html), and PhyML was used to construct respective phylogenetic trees [18]. PhyML was constructed using maximum likelihood with a bootstrap value of 1000 iterations, and all positions containing gaps and missing data were excluded in order to achieve phylogenetic trees. Finally, the trees were visualized and managed in iTOL [19]. ATGLCAT14A belongs to the B5 clade, while ATGLCAT14C belongs to B4.i clade

**Fig. 2:**
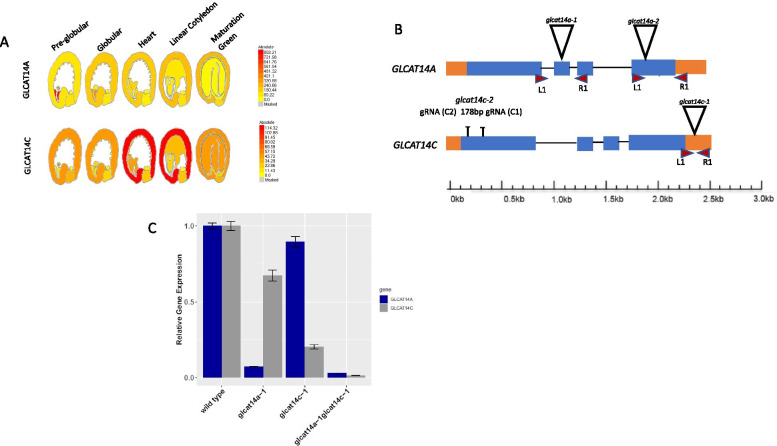
Overview of *GLCAT14A* and *GLCAT14C* gene expression and mutations. **a** *ATGLCAT14A* and *ATGLCAT14C* are expressed in the seed coat [14] **b** T-DNA insertions (inverted triangles), CRISPR mutants (lines) and qRT-PCR primers (red arrowheads) are indicated. The orange rectangles represent the UTRs; the blue rectangles represent the exon coding regions, while the lines between the exons represent the introns. **c** *GLCAT14A* and *GLCAT14C* gene expression in WT and *glcat14* mutant siliques at linear cotyledon stage (8 DAP). Transcript levels were normalized to the mean of one reference gene, the Arabidopsis actin 2 gene, *AtACT2*. Averages of three biological replicates ± SE are shown. Asterisks indicate significant differences compared with wild type (Student’s *t* test, *P* < 0.01)

GLCATs are involved in the transfer of GlcA to type II AG glycans. Although eleven confirmed and/or putative β-GLCATs have been identified in Arabidopsis, phylogenetic analysis showed that *GLCAT14A* (*AT5G39990*) and *GLCAT14B* *(AT5G15050*) appear to be paralogs, while *GLCAT14C (AT2G37585)* is phylogenetically distinct from *GLCAT14A* and *GLCAT14B* (Fig. 1). Seed microarray data displayed by the eFP browser [14, 15] revealed that *GLCAT14A* and *GLCAT14C* had elevated expression in the seed coat (Supplemental Fig. 1) and in seed development, primarily during the heart and linear cotyledon stages (Fig. 2a). To this end, we examined *glcat14a-1* and *glcat14c-1* single mutants and a *glcat14a-1glcat14c-1* double mutant to reveal the role of these genes in seed mucilage biosynthesis.

Single mutants (*glcat14a-1* and *glcat14c-1*) and the double mutant (*glcat14a-1glcat14c-1*) were examined for the expression of *GLCAT14A* and *GLCAT14C* using quantitative reverse transcription (qRT)-PCR. Given the expression of *GLCAT14A* and *GLCAT14C* across seed developmental stages (Fig. 2a), we examined their expression at the linear cotyledon stage (8 DAP) in wild type (WT), *glcat14a-1*, *glcat14c-1* and *glcat14a-1glcat14c-1* mutants, and observed a significant reduction in gene expression of *GLCAT14A* and *GLCAT14C* in both the single and double mutants (Fig. 2c). While we were unable to confirm the presence of a second T-DNA insertion in the SALK_051810 line for *glcat14c-2*, we did utilize a CRISPR knockout of the *GLCAT14C* gene close to its 5’ end that resulted in a 178 bp gene deletion, and produced similar phenotypes as the *glcat14c-1* (SALK_005705) mutant [20].

### The *glcat14a-1* and *glcat14a-1glcat14c-1* mutants have distinct seed coat mucilage phenotypes in response to different chemical extractants

**Fig. 3:**
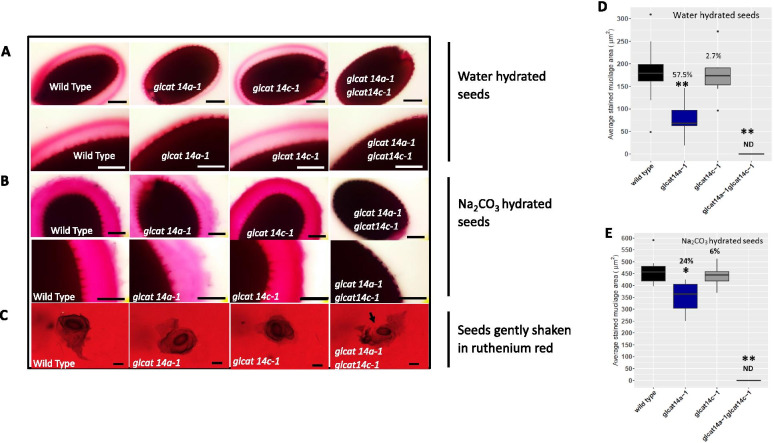
The *glcat14a-1* and *glcat14a-1glcat14c-1* mutants have seed mucilage defects. **a** Seeds hydrated in water were stained with 0.01% RR. **b** Seeds hydrated in 1 M Na_2_CO_3_ and stained with 0.01% RR. Loss of adherent mucilage was observed in the *glcat14a-1glcat14c-1* double mutant seeds coupled with the “peeling off” of the adherent layer from the seed coat when gently shaken, as indicated by an arrow (**c**). Note that in the WT and *glcat14* single mutants, the adherent layer surrounded the seed coat. Quantification of the average stained mucilage area for water hydrated seeds (**d**) and Na_2_CO_3_ hydrated seeds (**e**). Box plots were generated from three biological replicates of (> 20 seeds each). The percentage (%) decrease in mucilage area (**d** and **e**) were indicated for each mutant relative to the WT. The single and double asterisk marks a significant decrease compared with WT (Student’s *t-*test, *P* < 0.05 for single asterisks and *P* < 0.01 for double asterisks). ND- Not detected. Bars = 150 μm

**Fig. 4:**
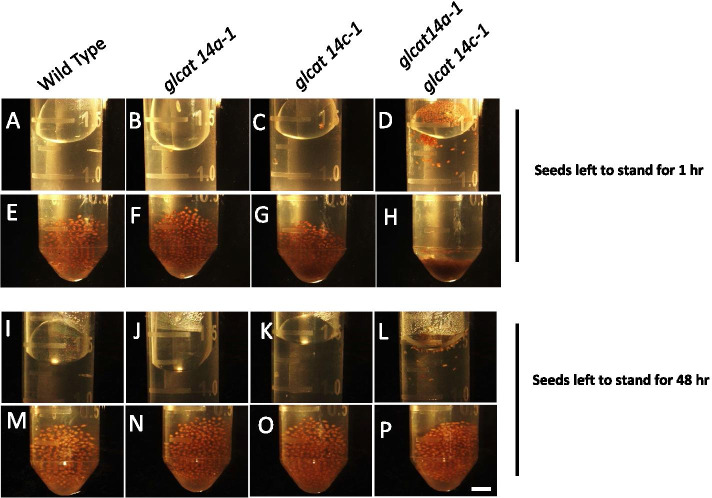
Seed floating and compactibility were displayed in *glcat14a-1glcat14c-1* mutants. Seeds of WT and mutants were shaken and left to stand for one hour (**a**-**f**) and 48 h (**i**-**p**). **a**-**d** and **i**-**l** represent the top layer of the sample tubes while E–F and M-P represent the lower part of the sample tubes. *glcat14a-1glcat14c-1* mutant seeds floated (**d**) and compacted (**h**) after being left to stand for 1 h and germinated after 48 h while staying afloat. Similar seed quantities were added to each tube. Bar = 0.75 mm

WT and mutant seeds were hydrated in distilled water and Na_2_CO_3_ and stained with ruthenium red (RR), a red dye which preferentially binds to unesterified pectin [21]. Seeds shaken in water and stained with RR showed that *glcat14a-1* seeds had a smaller mucilage capsule, while the adherent mucilage layer in *glcat14a-1glcat14c-1* mutant seeds was undetectable compared to WT (Fig. 3a). Similarly, the quantification of mucilage areas in hydrated seeds showed that relative to WT, *glcat14a-1* and *glcat14c-1* had a 57.5% and 2.7% reduction in mucilage area, respectively, while the mucilage area in *glcat14a-1glcat14c-1* could not be determined (Fig. 3d). Given the loss of adherent mucilage in the *glcat14a-1glcat14c-1* seeds (Supplemental Fig. 2D, H, L, N), it was unclear whether this mucilage deficient phenotype was due to mucilage extrusion defects or to the repartitioning of the mucilage layers. To answer this question, we investigated how the mutants extrude mucilage by dropping mature dry seeds in 0.01% RR dye. Results showed that *glcat14a-1glcat14c-1* seeds extruded mucilage like WT and single mutants, but then began “peeling off” the mucilage upon gentle shaking (Fig. 3c). While hydrating the seeds in water, the vast majority of the *glcat14a-1glcat14c-1* seeds floated (Fig. 4d), even after 1 h of extended contact with water. Also, the double mutant seeds were packed together (Fig. 4h), remained afloat and even germinated after 48 h (Fig. 4l).

Chemical extraction with Na_2_CO_3_ extracts pectins by cleavage of cross-linking ester linkages [22–24]. Treatment of the WT and single mutant seeds with 1 M Na_2_CO_3_ resulted in the rupturing of the cell wall to form organized ‘pyramidal’ arrangements of primary cell wall remnants attached to the columella, which was visualized as dark staining points on the seed surface. In *glcat14a-1glcat14c-1* seeds, the tangential and/or radial cell wall appears to be intact, lacking both the ‘pyramidal structure’ and the adherent mucilage (Fig. 3b). Similarly, the RR dye staining intensity of adherent mucilage was lower in *glcat14a-1* compared to WT, while the staining in *glcat14c-1* was indistinguishable from WT. Quantification of mucilage areas in mature seeds hydrated in 1 M Na_2_CO_3_ revealed that *glcat14a-1* and *glcat14c-1* mutants had a 24% and 6% reduction in mucilage area, respectively, while the mucilage area of *glcat14a-1glcat14c-1* seeds could not be determined due to the significant loss of adherent mucilage in the seed coat (Supplemental Fig. 3E-H; Fig. 3E).

### GLCAT14A and GLCAT14C influences cellulose ray morphology and cellulose deposition

**Fig. 5:**
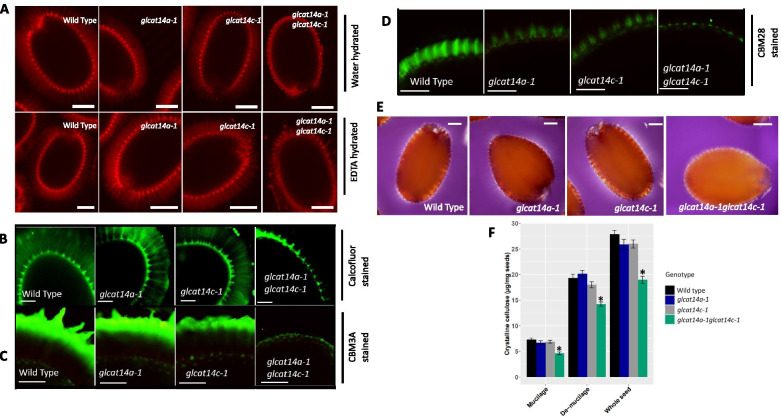
Cellulose deposition is altered in *glcat14a-1 glcat14c-1* double mutants. **a** Pontamine fast scarlet (S4B) cellulose staining of the adherent mucilage of water hydrated mature seeds (Upper panel) and EDTA hydrated seeds (lower panel). **b** Calcofluor staining of the adherent mucilage of WT, *glcat14* single and double mutant seeds. Immunolabelling of CBM3a (**c**) with high affinity to crystalline cellulose in adherent mucilage and CBM28 (**d**) were counterstained with the S4B dye. Visualization of polarized light birefringence by crystalline cellulose in adherent mucilage released from WT and mutant seeds (**e**). Quantification of crystalline cellulose contents in whole seeds, demucilaged seeds, and in the mucilage of WT and mutants (**f**) using the Updegraff assay. Values represents the means ± SD of 4 biological replicates. The single asterisk marks a significant decrease compared with WT (Student’s *t-*test, *P* < 0.05 for single asterisks). Bars = 100 μm

WT and *glcat14* mutant seeds were hydrated in distilled water and 50 mM EDTA and examined for the precise distribution of cellulose in the mucilage capsule using the S4B dye, which binds cellulose [25]. Results showed that WT and single mutant seed mucilage capsules displayed ordered and intense S4B-labeled cellulosic rays that projected outwards from the top of the columellae, as well as diffuse S4B signals between rays following water (Fig. 5a, Upper panel) and EDTA extractions (Fig. 5a, Lower panel). In contrast, *glcat14a-1glcat14c-1* seeds were characterized by irregular cellulose ray organization with incompletely detached primary cell walls in water hydrated seeds, and primary cell wall remnants bound tightly to the periphery of the extruded mucilage for EDTA hydrated seeds (Fig. 5a). To further characterize the fine structure and distribution of cellulose in seed adherent mucilage, we used calcofluor, a dye which binds β-glucans [26], and two carbohydrate-binding modules (CBMs; CBM3a and CBM28) immunolabelled in parallel with the S4B stain. CBM3a binds preferentially to crystalline cellulose structures, whereas CBM28 binds preferentially to amorphous cellulose structures [27]. Similar to the RR staining, we observed the loss of the feathery ray structure of the calcofluor stained adherent mucilage layer in the *glcat14a-1glcat14c-1* seeds compared to the WT (Fig. 5b, Supplemental Fig. 2N). In the WT and single mutants, CBM3a displayed a mustache tip-like structure that was concentrated especially at the outer periphery, whereas S4B stained the inner adherent layer and the rays above the columella. In contrast to WT*, glcat14a-1glcat14c-1* seeds had more severe defects as indicated by the absence of S4B stained ray-like structures, with some CBM3a immunolabelling detected at regions closest to the seed coat (Fig. 5c; Supplemental Fig. 3A). CBM28 labeling of *glcat14a-1* and *glcat14c-1* had a similar pattern as the WT but with reduced intensity; whereas, in *glcat14a-1glcat14c-1* double mutant, mucilage labeling was almost completely absent (Fig. 5d). Similarly, the adherent mucilage was observed for birefringence by any crystalline cellulose present and results indicated that WT and single mutant seeds showed bright regions with visible rays of crystalline cellulose within the adherent mucilage, but such birefringence was absent in *glcat14a-1glcat14c-1* seeds, except for the bright spots on the edges of seeds (Fig. 5e). Similarly, crystalline cellulose content in total mucilage, demucilaged and whole seeds showed that *glcat14a-1glcat14c-1* mutants had significantly reduced crystalline cellulose content relative to the wild type (Fig. 5f).

### Mucilage pectin components altered in *glcat14a-1* and highly altered in *glcat14a-1glcat14c-1*

**Fig. 6:**
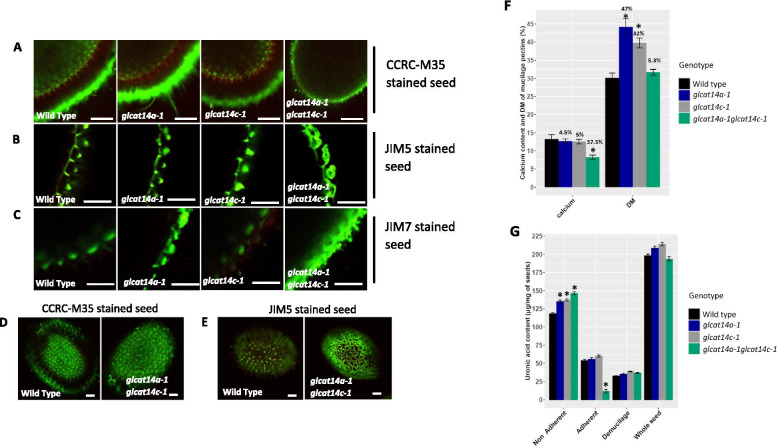
Pectin deposition is altered in *glcat14a-1* single mutants and *glcat14a-1 glcat14c-1* double mutants. Immunolabelling with CCRC-M35 (**a**, **d**), JIM5 (**b**, **e**) and JIM7 (**c**) of the adherent mucilage and counterstained with S4B. Biochemical determination of calcium content and degree of pectin methylesterification expressed as a percentage (**f**). Uronic acid estimation (**g**) was determined using the meta-hydroxydiphenyl method (see Methods). Percentage (%) increase or decrease relative to WT is indicated in F. Values represents the means ± SD of four biological replicates. The single asterisk indicates significant differences compared to WT (Student’s *t-*test, *P* < 0.05 for single asterisks). Bars = 100 μm; DM-degree of methylesterification

In addition to hydrating matured seeds in water and Na_2_CO_3_ and staining with RR, WT and *glcat14* mutant seeds were shaken in 50 mM EDTA to investigate whether there is any residual mucilage trapped in the seed coat. Typically, cation chelators like EDTA can facilitate mucilage extrusion by disrupting crosslinks in unesterified HG chains [28, 29]. Hydration of mature seeds in 50 mM EDTA, pH 8.0 showed that in contrast to the WT and two single mutants, *glcat14a-1glcat14c-1* double mutant seeds had primary cell wall remnants attached to the seed coat coupled with loss of adherent mucilage (Supplemental Fig. 4D and H). Given the reported role of glucuronic acid in calcium binding [12, 30], we investigated whether the addition of calcium ions impacts the pectic gel matrix of the adherent mucilage in *glcat14* mutant seeds. While the intensity of the RR stained mucilage of the *glcat14a-1* and *glcat14c-1* seeds shaken in 50 mM CaCl_2_ were comparable to the WT (Supplemental Fig. 4I-K, M-O), the RR staining intensity of *glcat14a-1glcat14c-1* adherent mucilage still displayed loss of adherent mucilage (Supplemental Fig. 4L, P and Q). Three pectin antibodies, JIM5 and JIM7, and CCRC-M35, were used in conjunction with S4B staining to examine the distribution of pectin relative to cellulose in the adherent mucilage. JIM5 and JIM7 are specific for partially methylesterified (up to 40%) and methyl esterified (up to 80%) HG respectively [31, 32], whereas CCRC-M35 recognizes unsubstituted RG-I backbones present in Arabidopsis seed mucilage [29, 33, 34]. CCRC-M35 labeling of WT and *glcat14* single mutant seeds appeared to surround the ray structures at the periphery of the mucilage halo (Supplemental Fig. 5A1-L1), whereas in the *glcat14a-1glcat14c-1* seeds, the CCRC-M35 labeling appeared to be at the surface of the seed coat and was not concentrated in a ray-like manner (Fig. 6a, d; Supplemental Fig. 5, panel J1-L1). Similarly, the distribution of partially methylesterified HG was also examined using the JIM5 antibody. Surprisingly, the diffuse JIM5 staining between columella present in the WT was absent in *glcat14a-1* and reduced in *glcat14c-1* mutant seeds (Supplemental Fig. 5, panel A2-I2). In contrast to WT, the *glcat14a-1glcat14c-1* seeds were intensely labeled at regions close to the columella, and at regions that appear to be incompletely detached primary cell wall fragments (Fig. 6b, e; Supplemental Fig. 5, panel J2-L2). Similar observations were made with JIM7 labelling with intense staining observed around the columella regions for *glcat14a-1* and *glcat14a-1glcat14c-1* (Fig. 6c; Supplemental Fig. 5, panel D3-F3 and J3-L3). To exclude the possibility that the changes in the HG esterification resulted from increased epitope accessibility, the calcium content and the degree of methylation (DM) of HG in the total mucilage extracts were determined using biochemical assays. Relative to WT, the calcium content decreased by 4.5%, 5% and 37.5% in *glcat14a-1*, *glcat14c-1* and *glcat14a-1glcat14c-1* mutants, respectively, while the DM of HG increased by 47%, 32% and 5.3% in *glcat14a-1*, *glcat14c-1* and *glcat14a-1glcat14c-1* mutants, respectively (Fig. 6f). Similarly, in contrast to WT, the uronic acid content of the non-adherent mucilage increased significantly in both the single and double mutants while a significant reduction was observed in the *glcat14a-1glcat14c-1* mutant in the adherent layer (Fig. 6g). The mucilage polymers were further assessed using immunoblot analyses and showed differences in polymer constituents between the WT and *glcat14* mutants (Supplemental Fig. 6A-C). While we observed increased CCRC-M35 epitope binding for *glcat14a-1* and *glcat14c-1* in the adherent mucilage, a significant reduction was observed in *glcat14a-1glcat14c-1* mutants relative to WT (Supplemental Fig. 6D). Notably, we found that JIM13 epitopes were detected and localized to the columella but we did not observe any difference in the JIM13 signal between WT and *glcat14* mutants (Supplemental Fig. 5, panels A4, D4, G4 and K4). Overall, our results obtained using biochemical and immunolabelling approaches provide evidence that the pectic organization in the seed coat mucilage is severely affected in *glcat14a-1glcat14c-1* mutants.

### Sugar distribution altered in *glcat14a-1glcat14c-1* mucilage

**Fig. 7:**
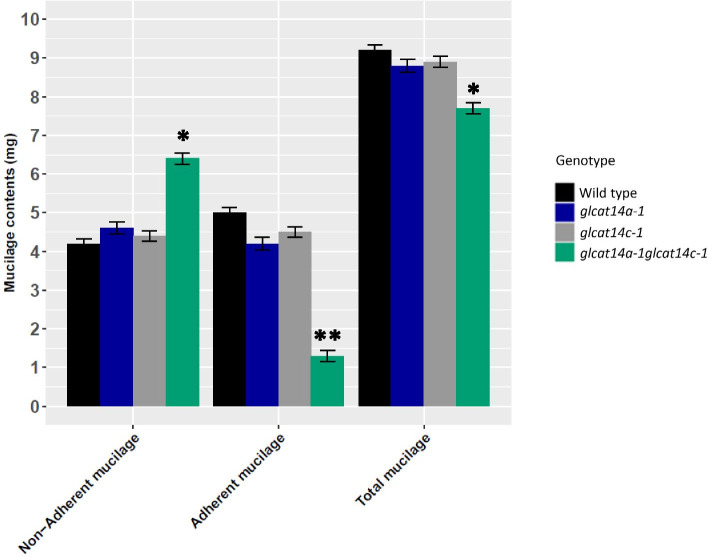
Mucilage weights of WT and *glcat14* mutants. The total mucilage is a combination of the adherent and the non-adherent mucilage. Significant alterations in mucilage content was observed in *glcat14a-1glcat14c-1* double mutants in comparison to WT. Values represents the means ± SD of three biological replicates. The asterisk indicates significant differences compared to WT (Student’s *t-*test, *P* < 0.05 for single asterisks, *P* < 0.01 for double asterisks)

The two most abundant sugars of the mucilage carbohydrates are rhamnose and galacturonic acid; together they make up approximately 80% of the total mucilage [5, 35]. To examine the effects of *glcat14a-1glcat14c-1* mutation had on mucilage composition, sugar analysis was performed. We observed a significant reduction in GlcA content (mol%) for *glcat14a-1glcat14c-1* mutants in the non-adherent mucilage relative to WT. Similarly, GlcA was not detected in the adherent mucilage of the *glcat14* mutants, only in the WT (Table 1). Although, we observed a slight increase in Gal and Xyl for *glcat14a-1glcat14c-1* seeds, these increases were not significant (P > 0.05). Compared to WT, *glcat14a-1* and *glcat14c-1* had significant alterations in the galacturonic acid (GalA) content in the mucilage layers, while the remaining sugars were comparatively similar to the WT. Surprisingly, the *glcat14a-1glcat14c-1* mutant showed a significant increase in GalA with a corresponding decrease in other sugars in the non-adherent mucilage layer, while in the adherent layer, we observed an increase in other sugars except GalA (Table 1). Also, we observed a significant reduction in the total mucilage content in *glcat14a-1glcat14c-1* relative to WT (Fig. 7). To further confirm the shift in the sugar composition of the mucilage layers, sequential extractions of WT and mutant seeds with ammonium oxalate, 0.2 N NaOH and 2 N NaOH showed a significant increase in the total sugar content for the *glcat14a-1glcat14c-1* mutant in ammonium oxalate and 0.2 N NaOH extracts, and a significant decrease in the 2 N NaOH extracts (adherent layer) (Table 2).

**Table 1:** Monosaccharide composition analysis of WT and *glcat14* mutants

Non-Adherent mucilage (mol %)					Adherent mucilage (mol%)				Whole Seed (mol%)			
Sugar	Wild Type	*glcat14a-1*	*glcat14c-1*	*glcat14a glcat14c*	Wild Type	*glcat14a-1*	*glcat14c-1*	*glcat14a glcat14c*	Wild Type	*glcat14a-1*	*glcat14c-1*	*glcat14a glcat14c*
Fuc	1.31 ± 0. 06	1.12 ± 0.02	1.05 ± 0.03	0.44 ± 0.01*	0.71 ± 0.04	0.82 ± 0.02	0.65 ± 0.04	2.66 ± 0.01**	2.02 ± 0.13	1.92 ± 0.14	1.62 ± 0.53	1.79 ± 0.36
Rha	31.53 ± 0.51	28.61 ± 0.17	27.44 ± 0.41	29.2 ± 0.39**	25.58 ± 0.43	27.85 ± 0.69	28.47 ± 0.41	26.63 ± 0.22**	17.03 ± 0.25	18.42 ± 0.24	18.03 ± 0.27	16.67 ± 0.54
Ara	0.38 ± 0.01	0.34 ± 0.05	0.29 ± 0.08	0.15 ± 0.01**	0.09 ± 0.012	0.12 ± 0.013	0.18 ± 0.015	0.60 ± 0.01**	20.59 ± 0.13	19.37 ± 0.37	20.24 ± 0.67	19.61 ± 0.46
Gal	3.68 ± 0.09	3.59 ± 0.09	4.97 ± 0.08	2.74 ± 0.05*	5.97 ± 0.02	6.09 ± 0.11	5.78 ± 0.04	13.20 ± 0.07**	15.76 ± 0.14	15.39 ± 0.45	15.81 ± 0.26	16.77 ± 0.32
Glc	3.80 ± 0.01	3.92 ± 0.03	3.63 ± 0.02	2.28 ± 0.06*	5.87 ± 0.07	5.98 ± 0.06	6.36 ± 0.05	10.85 ± 0.06**	4.69 ± 0.12	5.16 ± 0.25	4.65 ± 0.12	6.72 ± 0.36*
Xyl	7.95 ± 0.01	7.55 ± 0.02	5.53 ± 0.02	5.4 ± 0.05*	4.8 ± 0.06	5.43 ± 0.22	4.55 ± 0.03	7.66 ± 0.08**	12.76 ± 0.23	12.03 ± 0.66	12.39 ± 0.37	13.04 ± 0.83
Man	3.42 ± 0.04	3.03 ± 0.03	2.68 ± 0.04	1.20 ± 0.03*	3.23 ± 0.25	2.89 ± 0.36	2.84 ± 0.17	6.53 ± 0.11**	2.76 ± 0.25	2.11 ± 0.11	2.00 ± 0.39	2.38 ± 0.82
GalA	47.59 ± 0.13	51.55 ± 0.68	54.15 ± 0.73*	58.55 ± 0.92**	53.7 ± 0.35	50.81 ± 0.67*	51.17 ± 0.98	31.87 ± 0.11**	24,39 ± 0.16	25.60 ± 0.45	25.25 ± 0.61	23.02 ± 0.69
GlcA	0.34 ± 0.01	0.30 ± 0.03	0.26 ± 0.03	0.04 ± 0. 01**	0.05 ± 0.01	**n.d**	**n.d**	**n.d**				

^*^P < 0.05; **P < 0.01; n.d Not detected; Sugars significantly different from WT are indicated in bold

**Table 2:** Total sugar estimation (μg/mg of seed) in WT and *glcat14* mutants

Extracts^a^	WT	*glcat14a-1*	*glcat14c-1*	*glcat14a-1glcat14c-1*
Ammonium oxalate	5.74 ± 0.46	6.03 ± 0.35	5.97 ± 0.12	7.80 ± 0.22*
0.2 N NaOH	11.73 ± 0.63	13.9 ± 0.42*	12.94 ± 0.83	15.04 ± 0.42*
2 N NaOH	10.59 ± 0.72	9.6 ± 0.32	10.23 ± 0.29	4.39 ± 0.33*
Total Sugar	28.06	29.53	29.14	27.23

^a^Quantification of total sugars from wild type and *glcat14* mutants mucilage sequentially extracted using 0.2% ammonium oxalate, 0.2 N NaOH and 2 N NaOH neutralized and assayed with the phenol–sulfuric acid method against glucose standards. Results are given as µg/mg seed ± SE. Significant differences from wild type (WT), (*P* < 0.05) are indicated with an asterisk and shown in bold

### GLCAT14A and GLCAT14C required for seed coat epidermal cell development

**Fig. 8:**
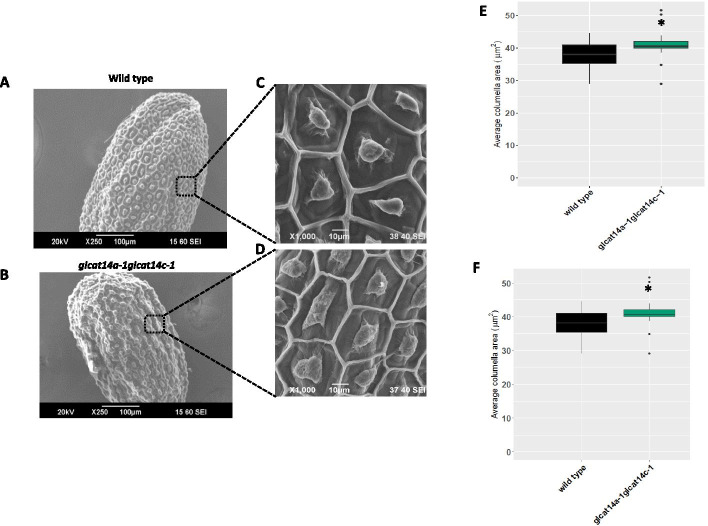
Columella and radial cell wall are severely impaired in the *glcat14a-1glcat14c-1* double mutant seeds. Scanning electron microscopy of the seed coat surface of WT (**a**) and *glcat14a-1glcat14c-1* double mutant seeds (**b**). Seeds were imbibed in water and visualized for alterations in the seed coat surface in WT (**c**) and *glcat14a-1glcat14c-1* double mutant seeds (**d**). Quantification of columella area before water hydration (**e**) and after water hydration (**f**). *, Significant differences from wild type (Student *t* test, *P* < 0.05)

**Fig. 9:**
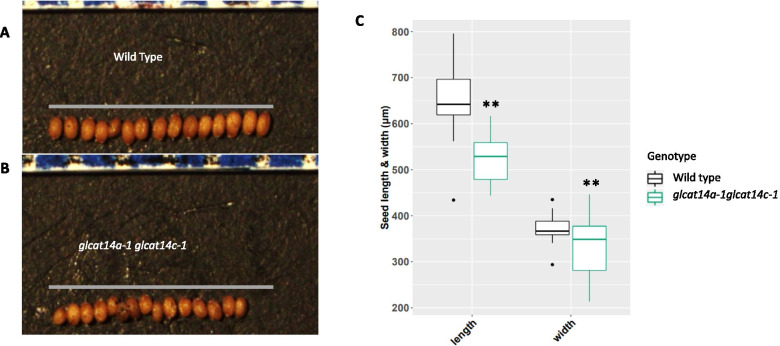
Seed size is reduced in the *glcat14a-1glcat14c-1* double mutant seeds. **a**, WT seeds were arranged horizontally and compared to *glcat14a-1glcat14c-1* double mutant seeds (**b**). Quantification of the length and width of WT and *glcat14a-1glcat14c-1* double mutant seeds (**c**), with the *glcat14a-1glcat14c-1* double mutant seeds displaying a significant reduction in seed size, *n* = 45 seeds. ** Significant differences from WT (Student *t* test, *P* < 0.01). Bar = 1 mm; Blue and white boarders above panel **a** and **b** are the measuring ruler

We employed SEM to observe any potential alterations in the SCE cells that are reflective of the changes in the cell wall polymer characteristics. While the surface morphology of the *glcat14a-1* and *glcat14c-1* seeds were indistinguishable from the WT (Supplemental Fig. 7), the surface morphology of the *glcat14a-1glcat14c-1* seeds displayed alteration of the SCE cells characterized by morphostructural changes in the radial cell walls of the hexagonal plane (Fig. 8a, and b), coupled with changes in the appearance of the columella after water imbibition (Fig. 8c, and d). Similarly, in contrast to WT, we observed a significant increase in the columella area of *glcat14a-1glcat14c-1* seeds before and after shaking the seeds in water (Fig. 8e and f). We also measured the seed size and found that *glcat14a-1glcat14c-1* seeds had significantly reduced seed length and width relative to WT (Fig. 9a, b and c).

## Discussion

Over the years, research efforts have been tailored towards identifying components of the mucilage polysaccharides that are important in the organization of the mucilage matrix. Despite the significant advances made in the discovery of the GTs involved in mucilage formation, accumulating evidence indicates that our understanding of the mucilage polysaccharide matrix formation and organization is far from complete. Here, we provide evidence that two *GLCAT* genes involved in the transfer of glucuronic acid to type II AGs in AGPs are critically important in controlling the structural integrity of the mucilage polysaccharide matrix and seed size, and elucidates the relationship between β-linked glucuronic acid residues in AGPs and the stability of the mucilage polysaccharide architecture.

### Loss of function of GLCAT14A and GLCAT14C results in seed flotation

Arabidopsis seeds, when imbibed in water, form a dense mucilage layer around the seed which makes them sink. Interestingly, the seed floating phenotype displayed by the *glcat14a-1glcat14c-1* seeds phenocopies previously characterized mucilage mutants of the floating mucilage-releasing (FMR) natural Arabidopsis accessions. The genes responsible for the FMR defects were speculated to be involved in cellulose formation given the absence of cellulose labelling on the adherent layers in FMR mutant seeds [36]. A similar seed floating phenotype was observed in *irx14* mutants with impaired xylan synthesis [37], suggesting that in addition to the reduction of cellulose, the absence of other mucilage polymers can also contribute to the FMR phenotype. Our results show that the *glcat14a-1glcat14c-1* seeds displayed phenotypes similar to those demonstrated by the *irx14* mutant [37] and FMR natural Arabidopsis accessions [36], and extends our previous knowledge on how intricately intertwined and interdependent the matrix polymers are, and how the genetic disruption of one of the interacting partners can have a profound effect on mucilage matrix architecture. It is worth noting that seed floating phenotypes have also resulted from impaired mucilage release [2, 38], but that was not the case for the *glcat14a-1glcat14c-1* seeds, which released mucilage upon contact with water-dissolved RR dye (Fig. 3c). This seed floating phenotype appears to be evolutionarily advantageous in improving seed dispersal over rivers, while still retaining its germination properties [37].

### Loss of function of GLCAT14A and GLCAT14C results in severe mucilage phenotypes

Several studies investigating the loss of function of genes/enzymes involved in mucilage formation have been characterized by the repartitioning of the mucilage layers upon water imbibition. In water hydrated seeds, we observed that *glcat14a-1* seeds displayed a significant reduction in adherent mucilage relative to WT, with a loss of adherent mucilage in *glcat14a-1glcat14c-1* seeds (Fig. 3a). The distinct mucilage phenotypes of *glcat14a-1* and *glcat14c-1* seeds may reflect the distinct mechanisms of action in the glucuronidation process that may be influenced by the glycan architecture based on the finding that ATGLCAT14A prefers β-1,6- galactans while ATGLCAT14C prefers β-1,3-galactans as substrates in an *in-vitro* assay [11]. Our observations thus reinforce the idea that GLCAT14A and GLCAT14C may perform distinct functions, as *glcat14a-1glcat14c-1* seeds lacking functional GLCAT14A and GLCAT14C exhibited much more severe phenotypes than either of the two single mutant seeds. Notably, a reduction of adherent mucilage was also observed in the CRISPR-Cas9 generated *glcat14abc* mutant [20] as revealed by staining with RR.

Given the striking mucilage phenotype of *glcat14a-1glcat14c-1* seeds in water, we tested for potential mucilage extrusion defects by hydrating seeds in 50 mM EDTA pH 8.0 and stainning in RR dye, given that cation chelators like EDTA can disrupt cross-linked unesterified HG chains and facilitate mucilage extrusion [2, 28]. Interestingly, the mucilage capsules of both *glcat14a-1* and *glcat14c-1* were similar to WT (Supplemental Fig. 4A-C, E-G); but surprisingly, the *glcat14a-1glcat14c-1* double mutant seeds lacked any detectable adherent mucilage (Supplemental Fig. 4D, H), suggesting that the mucilage phenotype characteristic of *glcat14a-1glcat14c-1* seeds was due to mucilage repartitioning and not to mucilage extrusion defects.

In addition to the calcium crosslinking of HG to form the “egg-box” structure, Ca^2+^-driven cross-linking among carboxyl groups of the uronic acid residues within the AGPs and the pectic acids have been speculated [39, 40]. With increased Ca^2+^ ion concentration contributing to mucilage adherence in the adherent layer [41], we analyzed RR stainings of *glcat14* mutant seeds treated with CaCl_2._ Despite the inherent loss of adherent mucilage in CaCl_2_ treated *glcat14a-1glcat14c-1* mutant seeds (Supplemental Fig. 4L, P), we cannot rule out the possibility that the loss of adherent mucilage mediated by the loss of GlcA residues might have been further excercebated by a reduction of calcium [12] and thus may contribute to the mucilage defect observed in *glcat14a-1glcat14c-1* mutant seeds.

### Both GLCAT14A and GLCAT14C are required for mucilage matrix polymer organization and assembly

Several studies directed at understanding the molecular mechanisms involved in mucilage formation have identified some key players involved in mucilage polysaccharide formation [1]. SOS5/FLA4, the only AGP extensively characterized to date to be involved in mucilage formation, has been implicated in maintaining cell wall structure [42–44], and required for mucilage adherence and formation of ray structure [35]. As GlcA is the only acidic sugar in the type II AG glycan with a reported role in calcium binding [40], we demonstrated that GlcA is essential for pectin and cellulose matrix organization in Arabidopsis seed mucilage as revealed by immunolabelling and biochemical analyses. By labelling with calcofluor, a florescent probe for cellulose and other β-glucans, we showed the expected combination of intense rays emanating from the top of the columella and diffused staining of rays in the adherent layer for WT, *glcat14a-1* and *glcat14c-1* (Fig. 5b). In addition to the calcofluor labelling around the columella in *glcat14a-1glcat14c-1* seeds, the diffuse staining of the rays was completely absent. Similarly, in contrast to WT, *glcat14a-1 glcat14c-1* seeds stained with pontamine S4B displayed an irregular distribution of cellulose rays, reduced diffused ray staining between the rays and the incomplete detachment of the outer cell wall in EDTA and water imbibed seeds following pontamine S4B staining (Fig. 5a). Both *sos5* and *fei2* mutant seeds have some mucilage defects such as absence of cellulosic rays, but with intact diffuse staining [6, 35] that are similar to those observed in *glcat14a-1glcat14c-1* seeds. Previous genetic mutant analysis indicated that GALT2, GALT5, SOS5, FEI1, and FEI2 act in a linear, non-additive pathway and suggested that glycosylated SOS5 interacts with FEI1/FEI2 [45]. Calcium binding of glucuronidated AG polysaccharides in AGPs such as SOS5 may facilitate receptor-ligand interactions necessary for the activity of receptor-like kinases [12]. We speculate that the disruption of the *GLCAT14A* and *GLCAT14C* genes may interefere with such interactions and hence associated receptor-like kinase activity. Perhaps there are some AGPs in addition to SOS5/FLA4 that are involved in mucilage formation, and these mucilage AGPs may rely on GLCAT14A and GLCAT14C for biological activity. It is worth mentioning that although 85 AGPs have been identified as members of the superfamily of cell wall proteins [20], only SOS5 has been implicated in mucilage formation. Also, the reduction in GlcA (Table 1) may suggest the possible involvement of other GLCATs in mucilage organization and the precise roles of other GLCATs in mucilage formation remains to be elucidated.

Cellulose has been shown to play important roles in anchoring the adherent mucilage to the seed coat [44, 46]. Aligned cellulose microfibrils in crystalline cellulose produce birefringence of polarized light, and mutants with defects in crystalline cellulose content have been identified based on such altered birefringence [47]. The outer epidermal cells of WT and single mutant seeds exhibited strong birefringence with visible rays of crystalline cellulose within the adherent mucilage. By contrast, *glcat14a-1glcat14c-1* seeds displayed much less birefringence under polarized light, as evidenced by the bright spots on the edges of the seeds (Fig. 5e). This indicates that the crystalline cellulose content was reduced in *glcat14a-1glcat14c-1* mucilage and thus may have contributed to the loss of adherent mucilage. Understandably, crystalline cellulose was reduced in *cesa5* [46] and a similar observation was reported for *sos5* [44] and *irx14-1* [48]; but the observed effect of a decrease in β-GlcA content (Table 1) affecting crystalline cellulose content was rather unexpected. Our findings serve to illustrate how intricately intertwined mucilage polymers are and should be an important consideration in research efforts leading to the deconstruction of plant cell wall assembly processes.

Multiple lines of evidence have revealed potential interactions between type II AGs and pectin. For example, treatment of cell wall fractions with pectin-degrading enzymes allows for the increased release of AGPs [49, 50]. Similarly, AGPs have been shown to bind to pectins in a calcium-dependent manner [51]. Since glucuronic acid binds to calcium [40], and given the importance of calcium in HG crosslinking and esterification [41], the significant alteration in epitope binding of JIM5 (Fig. 6b; Supplemental Fig. 5A2-L2) and JIM7 (Fig. 6c; Supplemental Fig. 5A3-L3), especially in *glcat14a-1 glcat14c-1* mutants indicates that the loss of function of ATGLCAT14A and ATGLCAT14C results in drastic changes in the HG esterification process. While we observed a reduction in calcium content, especially in the *glcat14a-1glcat14c-1* mutants, we only observed an increase in DM in *glcat14a-1* and *glcat14c-1* mutants in total mucilage extracts (Fig. 6f). Surprisingly, that was not the case for the *glcat14a-1glcat14c-1* mutants, as a significant decrease in calcium did not result in increase in mucilage pectin DM. However, we observed an intense staining of JIM5 and JIM7 tightly bound to the seed coat surface around the columella for *glcat14a-1glcat14c-1* mutants (Fig. 6b and c). Unfortunately, we were unable to detect LM2, MAC207 and JIM8 epitopes during whole seed immunolabelling and immunoblotting experiments; however, JIM13 epitopes were detected and localized to the columella (Supplemental Fig. 5A4-L4). JIM13 is a monoclonal antibody that detects an AGP-related glycan, specifically the epitope: β-d-GlcA-(1,3)-α-d-GalA-(1,2)-α-l-Rha; [52]. This finding lends credence to an earlier observation that showed the prescence of AGPs in mucilage [35]. Given the reported role of glucuronic acid of AGPs in stabilizing the covalent attachment of rhamnosyl residues of pectin RG-I backbone to AGPs in APAP1 [10], the reduction or loss of GlcA residues in AGPs may have contributed to the loosely held mucilage of the adherent layer in *glcat14a-1glcat14c-1* mutants being released upon gentle shaking in water (Fig. 3c).

### Role of GLCAT14A and GLCAT14C in seed coat epidermal cell development

**Fig. 10:**
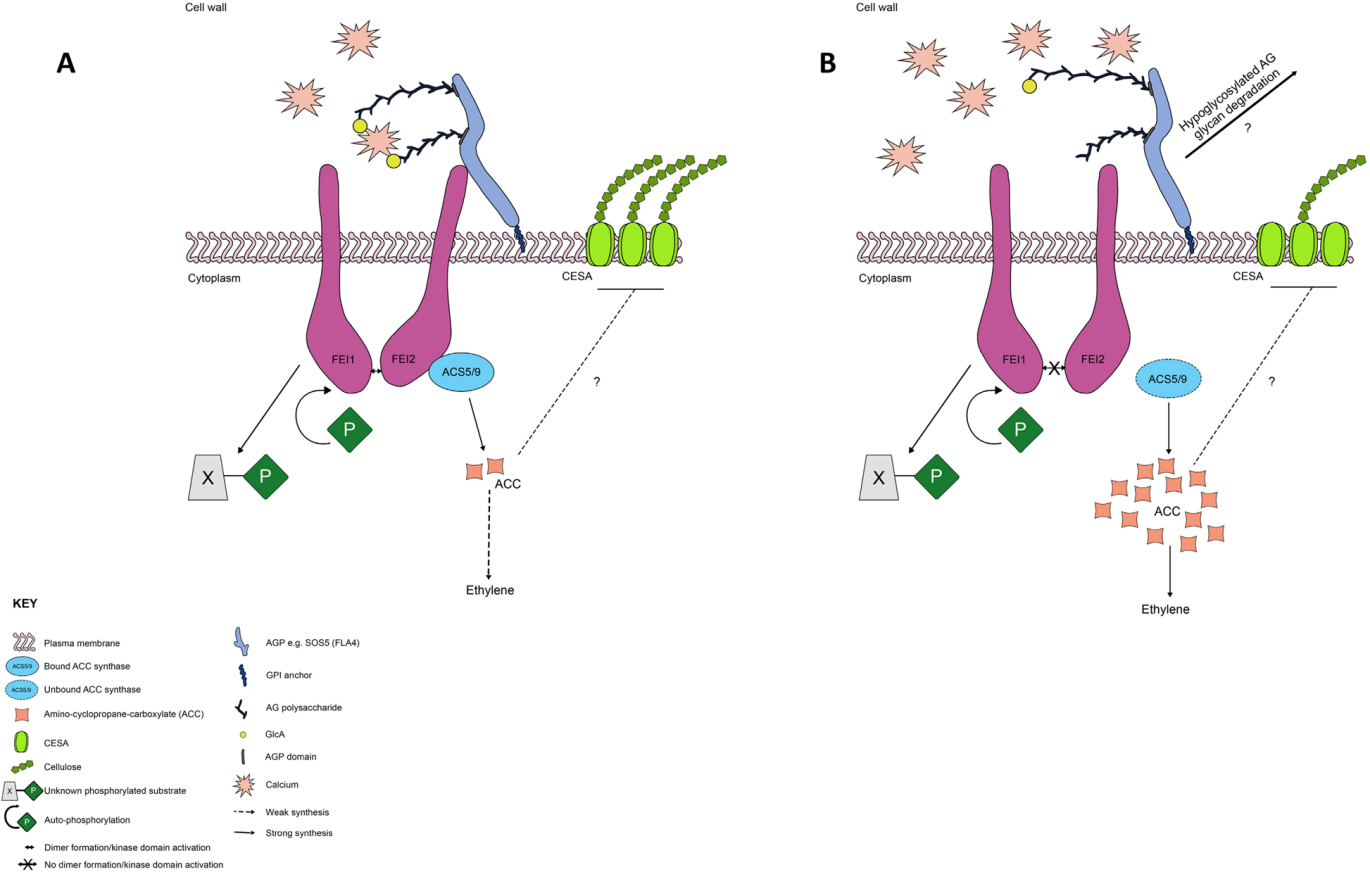
Proposed model for the potential role of ATGLCAT14A and ATGLCAT14C in Arabidopsis mucilage secretory cells during mucilage formation. This proposed model was based on previous studies [12, 42, 45, 56]. In **a**, the interaction of glucuronidated AG glycan and Ca^2+^ may stabilize the interaction between AGPs (e.g., SOS5) and FEI receptor kinases leading to the activation of the FEI kinase domain. FEI1/FE2 binding to ACC synthase (ACS5/9) limits the production of ACC, and influences either directly or indirectly, the formation of cellulose microfibril assembly, independent of ethylene [45]. In **b**, the absence or reduction of glucuronidated AG glycans may interfere with and reduce Ca^2+^ binding [12, 40] and may destabilize the activities of FEI receptor like proteins by altering potential interactions between AGPs and FEI. ACC synthase remains unbound to FEI proteins and becomes freely available for the increased production of ACC, which inhibits cellulose synthesis and increases the production of ethylene [42]. Similarly, hypoglycosylated moieties characterized by AG glycan modifications are targeted for destruction to the vacuoles via multivesicular bodies [56]

The surface morphology of mature WT and *glcat14a-1glcat14c-1* seeds were examined by SEM to investigate whether β-GlcA has a role in seed coat epidermal (SCE) cell development. We observed that SCE cells in *glcat14a-1glcat14c-1* seeds were deformed with the collapse of their polygonal structures (Fig. 8a and b). Specifically, we observed a collapse of the radial cell wall coupled with an increase in the size of the columella (Fig. 8c-f). Western et al*.* [53] suggested that a decrease in the amount of mucilage synthesized results from a smaller mucilage pocket and a much flatter columella. That appears to be the case here, as our data showed an increase in columella size before mucilage extrusion and remains unchanged following mucilage extrusion. This might explain the reason for the reduction in total mucilage content for the *glcat14a-1glcat14c-1* mutant (Fig. 7) advanced by smaller mucilage pockets that are known to precede columella formation [35]. Evidently, CESA2, CESA5 and CESA9 are involved in radial cell wall reinforcement and columella deposition [54, 55], but it remains to be determined whether the reduction in crystalline cellulose content in *glcat14a-1glcat14c-1* seeds might have impacted the columella formation. Notably, fully glycosylated FLA4/SOS5 molecules were identified to be candidates transported to the plasma membrane while insufficiently *O*-glycosylated protein regions are targeted for vacuolar degradation [56]. In that case, the modification of SOS5 glycans (i.e., the loss of glucuronic acid) might have interfered with SOS5’s intracellular trafficking [56] and its proposed interactions with the FEI ectopic domain thus affecting mucilage polymer assembly and SCE cell formation (Fig. 10). We observed a significant reduction in seed size for *glcat14a-1glcat14c-1* mutants (Fig. 9a-c) relative to WT. Although an increase in Gal was observed for *glcat14a-1glcat14c-1*seeds relative to WT, this increase was found not to be significant. Therefore, further analysis is needed to validate the plausible hypothesis that β-GlcA terminates the elongation of β-(1 → 6)-galactan side chains [57].

## Conclusions

We have characterized two β-glucuronosyltransferases, GLCAT14A and GLCAT14C, and demonstrated their involvement in the maintenance of seed mucilage polysaccharide matrix organization in *Arabidopsis thaliana*. While the genetic knockout of *GLCAT14A* and *GLCAT14C* did not result in the total loss of GlcA residues in seed coat AGPs, the contributory roles of other *GLCAT* genes in the seed mucilage remains to be determined. Also, the potential involvement of GLCAT14A/C in the GALT2GALT5/SOS5/FEI1FEI2 pathway as it relates to cell wall function remains to be investigated. Our findings here add to the list of genes that are critical to seed mucilage biosynthesis. Future investigations into the biochemistry involved in cell wall polymer interactions in the seed coat will further increase our understanding of the underlying mechanistic processes involved in mucilage assembly and seed coat development.

## Methods

### Plant lines and plant growth conditions

Arabidopsis thaliana accession Columbia-0 (Col-0) and two T-DNA insertion lines for *At5g39990*-(*glcat14a-1*, Salk_064313 and *glcat14a-2*, Salk_043905) and *At2g37585*- (*glcat14c-1*; Salk_005705) were obtained from the Arabidopsis Biological Resource Center (ABRC, Ohio State University). Seeds were germinated on plates with 0.5% MS media, after 4 days of stratification in the dark at 4 °C and were grown under long-day conditions (16 h of light/8 h of dark, 22 °C, 60% humidity) in growth chambers. Seedlings were transplanted after 7 days and grown under long-day conditions (16 h of light/8 h of dark, 22 °C, 60% humidity). The *glcat14a-1glcat14c-1* double mutant was isolated from an F2 population from a cross between the two respective single-mutant parents.

### Mutant confirmation by PCR and qRT-PCR

Mutant plants were genotyped following DNA extraction using the 2 × CTAB method by utilizing gene-specific primers in conjunction with the LBb1.3 insert-specific primer (Table S1) targeting specific regions as indicated in Fig. 2b in a PCR. To analyze transcript levels of *GLCAT14A* and *GLCAT14C* in mutants, total RNA was extracted from siliques at the linear cotyledon stage (8 DAP). RNA (1 μg) was used for first-strand cDNA synthesis along with an oligonucleotide (dT20) primer and SuperScript III reverse transcriptase (Thermo Scientific). The qPCR was performed using appropriate qPCR primers (Table S1) following procedures described here [20]. Moreover, *GLCAT14A* and *GLCAT14C* expression during seed coat development was also examined using the Arabidopsis seed coat-specific expression browser (http://bar.utoronto.ca/efp_seedcoat/cgi-bin/efpWeb.cgi).

### Determination of Mucilage content

Three independent samples of 100 mg seeds of wild type, single mutants and double mutants were precisely weighed and extracted by vigorously shaking in 1 mL of distilled water for 5 min to isolate the non-adherent mucilage. The supernatants were completely transferred to separate tubes. One mL of distilled water was added to the remaining seeds and treated ultrasonically for 20 s [58] at room temperature using a Sonic Dismembrator Model 100 with the probe intensity set to 1. Supernatants were transferred to Eppendorf tubes to form the non-adherent mucilage. Both the non-adherent and the adherent mucilage contents were freeze dried and weighed to determine the mucilage content.

### Microscopy and Image Analysis

#### Ruthenium Red Staining and Quantification of mucilage area

Mature dry seeds of wild type and mutants were hydrated in distilled water, 50 mM CaCl_2_, 50 mM EDTA, pH 8.0 and 1 M Na_2_CO_3_ for 30 min, washed with water and then stained with the ruthenium red (RR) dye for 30 min at room temperature using 0.01% RR (Sigma, St Louis, MO, USA) as described elsewhere [26]. Mature seeds prehydrated in distilled water was stained using 25 μg/ml fluorescent brightener 28 (Sigma) for, 20 min at room temperature as previously described [26]. In both cases, seeds were shaken with a rotator and ruthenium red stained seeds were photographed using a Nikon SMZ1500 stereomicroscope coupled with a CCD Infinity 2 camera, while calcofluor stained seeds were imaged using a Zeiss LSM 510 confocal microscope. Pontamine staining of mature seeds hydrated in water and 50 mM EDTA were carried out as described earlier [25] using 0.01% pontamine fast scarlet S4B (Sigma) in 50 mM NaCl for 30 min. Seeds were then de-stained four times with water before examination using a confocal microscope. Dry mature seeds were dropped in a 12-well plate containing 0.01% Ruthenium red stain without shaking and after shaking very briefly, and images were acquired using a light microscope. The ruthenium red-stained mucilage area was quantified using FIJI (ImageJ) as described previously [59]. Regions of interest (ROI) were segmented in Fiji, and areas for the ROI were measured using the Analyze Particles function. Mucilage area was obtained by subtracting Seed area from Seed + Mucilage. Evaluation of statistical significance was conducted using R program by Tukey–Kramer HSD (*P* < 0.05).

### Immunohistochemistry

Whole-seed immunolabeling was conducted according to a published method, except that seeds were shaken in water before immunolabeling and that seeds were stained with S4B after immunolabeling [60]. Briefly, mature dry seeds were shaken in phosphate-buffered saline (PBS), pH 7.4 for 1 h. The supernatant (containing soluble mucilage components) was removed, and the remaining seeds with tightly bound mucilage were processed for immuno-fluorescence as follows: Seeds were shaken in 5% BSA in PBS for 30 min, washed with PBS, and incubated with the primary antibody CCRC-M35 [34] diluted 1/10 in 1% BSA in PBS for 1.5 h. Samples treated without a primary antibody served as a negative control. The specificities of the primary antibodies JIM5, JIM7, JIM13 and CCRC-M35 (CarboSource) have been extensively described [34]. CBM3a, mostly specific to crystalline cellulose, and CBM28, mostly specific to amorphous cellulose regions [27], were treated as primary antibodies in identical solutions before treatment with mouse anti-histidine (Qiagen). Goat anti-rat secondary antibody conjugated to AlexaFluor488 was used against JIM5, JIM7 and JIM13, whereas goat anti-mouse conjugated to AlexaFluor488 (Molecular Probes; Invitrogen) was used as a secondary and tertiary antibody against the CCRC-M35 and CBMs, diluted 1/100 in 1% BSA in PBS for 1.5 h. Immunolabelled seeds were counterstained with S4B [44] and imaged using a Zeiss LSM 510 confocal microscope. Signal intensities for each antibody treatment were preserved across genotypes; however, the signal intensity was varied between treatments. Confocal micrographs were further processed using imageJ [61]

### Enzyme-linked immunosorbent assay (ELISA) of CCRC-M35

Seeds (5 mg) of wild type, single mutants and double mutants were precisely weighed and extracted by vigorously shaking in 1 mL of distilled water for 5 min to isolate the non-adherent mucilage. The supernatants were completely transferred to separate tubes. One mL of distilled water was added to the remaining seeds and treated ultrasonically for 20 s [58] at room temperature using a Sonic Dismembrator Model 100 with the probe intensity set to 1. Supernatants were transferred to Eppendorf tubes to form the non-adherent mucilage. Two hundred (200 μl) of mucilage extracts were transferred to four wells on a 96-well ELISA plate (3598; Corning, Wiesbaden, Germany), while 200 μl of MilliQ water served as a negative control. The ELISA was carried out following methods described elsewhere [62], and the optical density (OD) value was read as the difference between the absorption value at 450 nm and 655 nm using a Synergy H1 microplate reader (BioTek, Bad Friedrichshall, Germany). The reading from each test well subtracted the value from the negative control well.

### Dot immunoblotting assays

Non-adherent and adherent mucilage extracts (1 mg/mL) were resuspended in water after freeze drying. A series of dilutions were prepared and a 1 μl aliquot was spotted onto a nitrocellulose membrane (Merck Millipore). After being air-dried, the membrane was blocked for 1 h in 3% BSA in PBS, and then it was incubated for 1.5 h in a tenfold dilution of primary antibodies. After washing three times with PBS, membranes were incubated for 1.5 h in horseradish peroxidase (HRP)-conjugated anti-rat (for JIM5 and JIM7) or anti-mouse (for CCRC M35) secondary antibodies in a 1000-fold dilution in in 1% BSA in PBS. Membranes were washed prior to color development in substrate solution [25 mL de-ionized water, 5 mL methanol containing 10 mg mL − 1, 4-chloro-1-naphthol and 30 μl 6% (v/v) H_2_O_2_]. After incubation for 30 min at room temperature, the blots were rinsed with de-ionized water and photographed.

### Scanning electron microscopy

Seed coat morphology was investigated using a JEOL JSM-6390 scanning electron microscope (Hitachi High-Technologies). Seeds were mounted on aluminum stubs using double adhesive tapestubs and sputter coated with a palladium alloy using a Cressington 208C high-resolution sputter coater (Ted Pella Inc.). Electron micrographs were processed and measured using imageJ [61].

### Determination of Monosaccharide Composition by HPAEC and total sugar content

Non-adherent and adherent mucilage and whole seed alcohol insoluble residue (AIR) extracts were carried out as described previously [58]. One hundred microliters of mucilage extracts (adherent and non-adherent) and 50 μl of 10 mg/mL AIR were transferred to glass tubes and were hydrolyzed using 2 N trifluoroacetic acid (TFA) at 121 °C for 90 min. TFA was removed by evaporation with N_2_ gas. Samples were dissolved in 500 μL milli-Q water containing 0.2 mM cellobiose as an internal standard. A standard sugar mixture (fucose, rhamnose, arabinose, galactose, glucose, xylose, mannose, galacturonic acid, and glucoronic acid) was used for making the standard curve. Monosaccharide compositions were calculated as molar percentages (mol %) and in absolute amounts (μg/mg of seeds). All samples and standards were subjected to high pH anion-exchange chromatography with pulsed amperometric detection (HPAEC-PAD) using a Dionex PA-20 column (Thermo Fisher Scientific, Sunnyvale, CA, USA) essentially as described here [63].

Total sugar (μg/mg seed) was determined by phenol–sulfuric assay [64] following sequential extraction with 0.2% ammonium oxalate, 0.2 N and then 2 N sodium hydroxide for 1 h each with vigorous shaking at 37 °C.

### Crystalline Cellulose Observation and Determination

For determination of crystalline cellulose content, 1 mL of distilled water was added to 10 mg of mature dry seeds and treated ultrasonically for 20 s as described previously [58] at room temperature. The supernatant was transferred into a separate tube, and the de-mucilaged seeds were kept for further analysis. Approximately, ten (10) milligrams of seeds (exact weight recorded) alongside the de-mucilage seeds were milled using steel balls for 5 min. AIR from de-mucilaged and whole seeds were isolated by two sequential washes with 1 mL of 70% (v/v) ethanol and centrifugation for 10 min at 13,200* g*. After washing the AIR extract with 1:1 (v/v) chloroform:methanol, followed by acetone, the pellet was dried for 5 min at 60 °C. Crystalline cellulose content was then determined as described previously [65], with minor modifications. The 2 mg of dry AIR (from whole and de-mucilaged seeds) together with 500 μl of the total mucilage extracted previously were mixed with 1 mL of Updegraff reagent (acetic acid:nitric acid:water, 8:1:2 [v/v]) before incubation at 100 °C for 30 min [65]. After hydrolysis, the Updegraff-resistant pellet (containing only crystalline cellulose) was rinsed once with water, once with acetone, dried, and then hydrolyzed using 200 μL of 72% (v/v) sulfuric acid. Crystalline cellulose amounts were quantified colorimetrically at 620 nm in a spectrophotometer using the anthrone reagent [65]. Seeds were also mounted in water on a microscope slide and observed with an epiflorescent microscope equipped with polarizing filters for birefringence by any crystalline cellulose in the investigated genotypes.

### Uronic acid estimation and biochemical determination of the Calcium and DM content of HG

Whole mucilage was extracted by shaking 20 mg mature dry seeds in 500 μL of distilled water using an ultrasonication treatment as described previously. For the degree of methylesterification (DM), 200μL of supernatant was transferred into a new tube and saponified with 0.25 M NaOH for 1 h at room temperature with tube rotation. The reaction was neutralized with 0.25 M HCl (to give a total volume of 600μL) and centrifuged for 10 min at 10,000 g. The amount of methanol released after the saponification reaction was measured by a colorimetric method [66]. Five hundred microliters of the supernatant was transferred into a new 1.5 mL tube, oxidized with 0.5 units of alcohol oxidase (Sigma-Aldrich) for 15 min at 25 °C, and incubated with 500 μL of freshly prepared 0.02 M 2,4-pentanedione (dissolved in 2 M ammonium acetate and 0.05 M acetic acid) for 15 min at 60 °C in a 1 mL total volume. After cooling on ice for 2 min, the absorbance was measured at 412 nm and quantified using a methanol standard curve.

The uronic acid content was determined by the meta-hydroxydiphenyl method [67] using GalA as the standard. One hundred μL of the saponified mucilage solution was transferred into a new 1.5 mL microcentrifuge tube, and hydrolysed with 1.2 mL of concentrated sulfuric acid containing 0.0125 M sodium tetraborate (Sigma-Aldrich) for 5 min at 100 °C. After samples were cooled on ice, 25 μL of 0.15% (w/v) meta-hydroxydiphenyl (Sigma-Aldrich) in 0.5% (w/v) NaOH was added and absorbance was measured at 525 nm. The DM of HG in the mucilage extracts were calculated as the percentage molar ratio of methanol to uronic acid [68]. Uronic acid content of de-mucilaged and whole seeds were also estimated from AIR samples using methods described previously [41]. For the estimation of calcium content, a commercial calcium colormetric assay kit (MAK022, Sigma-Aldrich, St. Louis, MO, USA) was used for calcium measurement following the manufacturer's protocol. Following mucilage extractions, calcium ions from the total mucilage extracts form a complex with the o-cresolphthalein in the assay kit, resulting in a color change from transparent to pink. The amount of calcium was determined using a UV spectrometer at OD_575_ and a standard curve made with different concentrations of CaCl_2_ and values were expressed as percentages.

## Supplementary Information

**Additional file 1: Supplemental Table 1.** List of primers used for mutant characterization. **Supplemental Figure 1.** Expression of the *GLCAT14A*, *GLCAT14B*, *GLCAT14C*, *FEI2* and *SOS5* genes in the Arabidopsis seed coat. The Arabidopsis eFP browser (http://bar.utoronto.ca/efp_seedcoat/cgi-bin/efpWeb.cgi) was used to examine the expression of the *GLCAT* genes involved in glucuronidation of Type II AG and was compared to the expression of the *FEI2* and *SOS5/FLA4* genes in the seed coat at 3 day post anthesis (DPA), 7 DPA and 11 DPA. **Supplementary Figure 2.** Mucilage phenotypes of WT and *glcat14* mutants hydrated in different chemical extractants. A-D**,** Staining of the adherent mucilage with 0.01% ruthenium red (RR) after vortexing briefly for 5 min in water (A-D), Na_2_CO_3_ (E-H) and 50mM CaCl_2_ (I-L).WT (M) and *glcat14a-1glcat14c-1* (N) seeds were shaken in water and stained with calcofluor, which primarily stains cellulose, but *also stains pectic galactan, xylan, and galactomannan to a lesser extent *[26]*.* Images (A-L) were acquired using a light microscope, while images M and N were acquired using a Zeiss confocal microscope using the same acquisition settings to acquire both images. Three independent experiments (each with more than 25 seeds) were performed with similar results. Bar = 200μm for (A-L); Bar = 100 μm (M and N). **Supplemental Figure 3.** Immunolabeling of crystalline and amorphous cellulose in WT and *glcat14* mucilage. Immunolabeling of crystalline cellulose with the CBM3a antibody (A) which binds preferentially to crystalline cellulose and the CBM28 antibody (B) which binds preferentially to amorphous cellulose in the adherent mucilage of the WT and *glcat14* mutants [27]. The cellulosic ray-structure was counterstained with the S4B dye (red fluorescence). Three independent experiments (each with more than 25 seeds) were performed and similar results were obtained in each case. All scale bars = 50 μm. **Supplementary Figure 4.** Mucilage phenotypes of WT and *glcat14* mutants. A-D, Staining of the adherent mucilage with 0.01% ruthenium red (RR) after vortexing briefly for 5 min in 50mM EDTA (A-H) and 50mM CaCl_2_ (I-P). E-H represents higher magnification of A-D, while M-P represents higher magnification of I-L. Lack of detectable adherent mucilage were observed for both EDTA (D and H) and CaCl_2_ imbibed (L and P) double mutant seeds. Addition of 50mM CaCl_2_ was unable to rescue the mucilage defect in *glcat14a-1 glcat14c-1* mutants seeds (L and P). Images (A-P) were acquired using light microscope with the same acquisition settings. Quantification of the average area of ruthenium red stained mucilage capsule for WT and *glcat14* mutant seeds hydrated in 50mM CaCl_2_(Q). The mucilage capsule is significantly reduced in *glcat14a-1* while *glcat14a-1glcat14c-1* could not be detected. Box plots were generated from 3 biological replicates of (>20 seeds each). The single and double asterisk marks a significant decrease compared with WT (Student’s *t-*test, P < 0.05 for single asterisks and P < 0.01 for double asterisks). ND- Not determined. Bar = 100μm. **Supplemental Figure 5.** Pectin immunolabeling in the WT and *glcat14* mucilage. Immunolabeling of WT and *glcat14* adherent mucilage with the CCRC-M35 antibody (A1-L1), JIM5 (A2-L2), JIM7 (A3-L3) and JIM13 (A4-L4) counterstained with S4B (red fluorescence). CCRC-M35 binds to unsubstituted rhamnogalacturonan I while JIM5 and JIM7 bind to partially methylesterified and highly methylesterified pectins [26], while JIM13 recognizes carbohydrate moieties associated with AGPs. Three independent experiments (each with more than 25 seeds) were performed and similar results were obtained. Scale bars = 50 μm, for A1-L1; Scale bars = 100 μm. **Supplemental Figure 6.** Pectin immunoblotting of extracted mucilage of WT and *glcat14* mutant seeds. Water-soluble and adherent mucilage was sequentially extracted from WT, *glcat14a-1*, *glcat14c-1*, *glcat14a-1glcat14c-1* seeds. Mucilage was diluted in a series of concentrations (for CCRC-M35 and JIM7) as specified prior to spotting on to nitrocellulose membrane. The membrane was hybridized with antibodies specifically binding to the unbranched RG-I backbone (CCRC-M35, A), and antibodies specific to pectin HG (JIM5, B and JIM7, C). CCRC-M35 enzyme-linked immunosorbent assay (ELISA) of non-adherent and adherent mucilage showed increased CCRC-M35 epitopes for *glcat14a-1* and *glcat14c-1* mutants, and a reduced epitope binding for *glcat14a-1glcat14c-1* double mutants. The y-axis denotes the CCRC-M35 ELISA-corrected absorbance. **Supplemental Figure 7.** Scanning electron microscopy of wild type, *glcat14a-1* and *glcat14c-1* seeds. Scanning electron microscopy of the seed coat surface of *glcat14a-1* and *glcat14c-1* mutant seeds are comparable to the wild type.

## Data Availability

The datasets used and /or analyzed during the current study are available from the corresponding author upon reasonable request.
