# Supplementary material for: Two β-glucuronosyltransferases involved in the biosynthesis of type II arabinogalactans function in mucilage polysaccharide matrix organization in Arabidopsis thaliana
Source: BMC Plant Biol. 2021 May 29;21:245. doi: 10.1186/s12870-021-03012-7 (PMC8164333; doi:10.1186/s12870-021-03012-7)
Supplement: Supplementary file 1 — Additional file 1: Supplemental Table 1. List of primers used for mutant characterization. Supplemental Figure 1. Expression of the GLCAT14A, GLCAT14B, GLCAT14C, FEI2 and SOS5 genes in the Arabidopsis seed coat. The Arabidopsis eFP browser (http://bar.utoronto.ca/efp_seedcoat/cgi-bin/efpWeb.cgi) was used to examine the expression of the GLCAT genes involved in glucuronidation of Type II AG and was compared to the expression of the FEI2 and SOS5/FLA4 genes in the seed coat at 3 day post anthesis (DPA), 7 DPA and 11 DPA. Supplementary Figure 2. Mucilage phenotypes of WT and glcat14 mutants hydrated in different chemical extractants. A-D, Staining of the adherent mucilage with 0.01% ruthenium red (RR) after vortexing briefly for 5 min in water (A-D), Na2CO3 (E-H) and 50mM CaCl2 (I-L).WT (M) and glcat14a-1glcat14c-1 (N) seeds were shaken in water and stained with calcofluor, which primarily stains cellulose, but also stains pectic galactan, xylan, and galactomannan to a lesser extent [26]. Images (A-L) were acquired using a light microscope, while images M and N were acquired using a Zeiss confocal microscope using the same acquisition settings to acquire both images. Three independent experiments (each with more than 25 seeds) were performed with similar results. Bar = 200μm for (A-L); Bar = 100 μm (M and N). Supplemental Figure 3. Immunolabeling of crystalline and amorphous cellulose in WT and glcat14 mucilage. Immunolabeling of crystalline cellulose with the CBM3a antibody (A) which binds preferentially to crystalline cellulose and the CBM28 antibody (B) which binds preferentially to amorphous cellulose in the adherent mucilage of the WT and glcat14 mutants [27]. The cellulosic ray-structure was counterstained with the S4B dye (red fluorescence). Three independent experiments (each with more than 25 seeds) were performed and similar results were obtained in each case. All scale bars = 50 μm. Supplementary Figure 4. Mucilage phenotypes of WT and glcat14 mutants. A-D, [file 12870_2021_3012_MOESM1_ESM.zip › Additional File 1.docx]

**Additional File 1: Supplemental Table 1. List of primers used for mutant characterization. Supplemental Figure 1. Expression of the *GLCAT14A*, *GLCAT14B*, *GLCAT14C*, *FEI2* and *SOS5* genes in the Arabidopsis seed coat.** The Arabidopsis eFP browser (<http://bar.utoronto.ca/efp_seedcoat/cgi-bin/efpWeb.cgi>) was used to examine the expression of the *GLCAT* genes involved in glucuronidation of Type II AG and was compared to the expression of the *FEI2* and *SOS5/FLA4* genes in the seed coat at 3 day post anthesis (DPA), 7 DPA and 11 DPA. **Supplementary Figure 2. Mucilage phenotypes of WT and *glcat14* mutants hydrated in different chemical extractants. A-D,** Staining of the adherent mucilage with 0.01% ruthenium red (RR) after vortexing briefly for 5 min in water (A-D), Na_2_CO_3_ (E-H) and 50mM CaCl_2_ (I-L).WT (M) and *glcat14a-1glcat14c-1* (N) seeds were shaken in water and stained with calcofluor, which primarily stains cellulose, but also stains pectic galactan, xylan, and galactomannan to a lesser extent [26]. Images (A-L) were acquired using light microscope, while images M and N were acquired using a Zeiss confocal microscope using the same acquisition settings to acquire both images. Three independent experiments (each with more than 25 seeds) were performed with similar results. Bar = 200µm for (A-L); Bar = 100 µm (M and N). **Supplemental Figure 3. Immunolabeling of crystalline and amorphous cellulose in WT and *glcat14* mucilage.** Immunolabeling of crystalline cellulose with the CBM3a antibody (A) which binds preferentially to crystalline cellulose and the CBM28 antibody (B) which binds preferentially to amorphous cellulose in the adherent mucilage of the WT and *glcat14* mutants [27]. The cellulosic ray-structure was counterstained with the S4B dye (red fluorescence). Three independent experiments (each with more than 25 seeds) were performed and similar results were obtained in each case. All scale bars = 50 μm. **Supplementary Figure 4. Mucilage phenotypes of WT and *glcat14* mutants.** A-D, Staining of the adherent mucilage with 0.01% ruthenium red (RR) after vortexing briefly for 5 mins in 50mM EDTA (A-H) and 50mM CaCl_2_ (I-P). E-H represents higher magnification of A-D, while M-P represents higher magnification of I-L. Lack of detectable adherent mucilage were observed for both EDTA (D and H) and CaCl_2_ imbibed (L and P) double mutant seeds. Addition of 50mM CaCl_2_ was unable to rescue the mucilage defect in *glcat14a-1 glcat14c-1* mutants seeds (L and P). Images (A-P) were acquired using light microscope with the same acquisition settings. Quantification of the average area of ruthenium red stained mucilage capsule for WT and *glcat14* mutant seeds hydrated in 50mM CaCl_2_(Q). The mucilage capsule is significantly reduced in *glcat14a-1* while *glcat14a-1glcat14c-1* could not be detected. Box plots were generated from 3 biological replicates of (>20 seeds each). The single and double asterisk marks a significant decrease compared with WT (Student’s *t-*test, P < 0.05 for single asterisks and P < 0.01 for double asterisks). ND- Not determined. Bar = 100µm. **Supplemental Figure 5. Pectin immunolabeling in the WT and *glcat14* mucilage.** Immunolabeling of WT and *glcat14* adherent mucilage with the CCRC-M35 antibody (A1-L1), JIM5 (A2-L2), JIM7 (A3-L3) and JIM13 (A4-L4) counterstained with S4B (red fluorescence). CCRC-M35 binds to unsubstituted rhamnogalacturonan I while JIM5 and JIM7 bind to partially methylesterified and highly methylesterified pectins [26], while JIM13 recognizes carbohydrate moieties associated with AGPs. Three independent experiments (each with more than 25 seeds) were performed and similar results were obtained. Scale bars = 50 μm, for A1-L1; Scale bars = 100 μm. **Supplemental Figure 6. Pectin immunoblotting of extracted mucilage of WT and *glcat14* mutant seeds.** Water-soluble and adherent mucilage was sequentially extracted from WT, *glcat14a-1*, *glcat14c-1*, *glcat14a-1glcat14c-1* seeds. Mucilage was diluted in a series of concentrations (for CCRC-M35 and JIM7) as specified prior to spotting on to nitrocellulose membrane. The membrane was hybridized with antibodies specifically binding to the unbranched RG I backbone (CCRC-M35, A), and antibodies specific to pectin HG (JIM5, B and JIM7, C).CCRC-M35 enzyme-linked immunosorbent assay (ELISA) of non-adherent and adherent mucilage showed increased CCRC-M35 epitopes for *glcat14a- 1* and *glcat14c-1* mutants, and a reduced epitope binding for *glcat14a-glcat14c-1* double mutants. The y-axis denotes the CCRC-M35 ELISA-corrected absorbance. **Supplemental Figure 7. Scanning electron microscopy of wild type, *glcat14a-1* and *glcat14c-1* seeds.** Scanning electron microscopy of the seed coat surface of *glcat14a-1* and *glcat14c-1* mutant seeds are comparable to the wild type.

**Supplementary Table 1: List of primers used for mutant characterization**
