# Supplementary material for: Two β-glucuronosyltransferases involved in the biosynthesis of type II arabinogalactans function in mucilage polysaccharide matrix organization in Arabidopsis thaliana
Source: BMC Plant Biol. 2021 May 29;21:245. doi: 10.1186/s12870-021-03012-7 (PMC8164333; doi:10.1186/s12870-021-03012-7)

Supplementary Figure 1

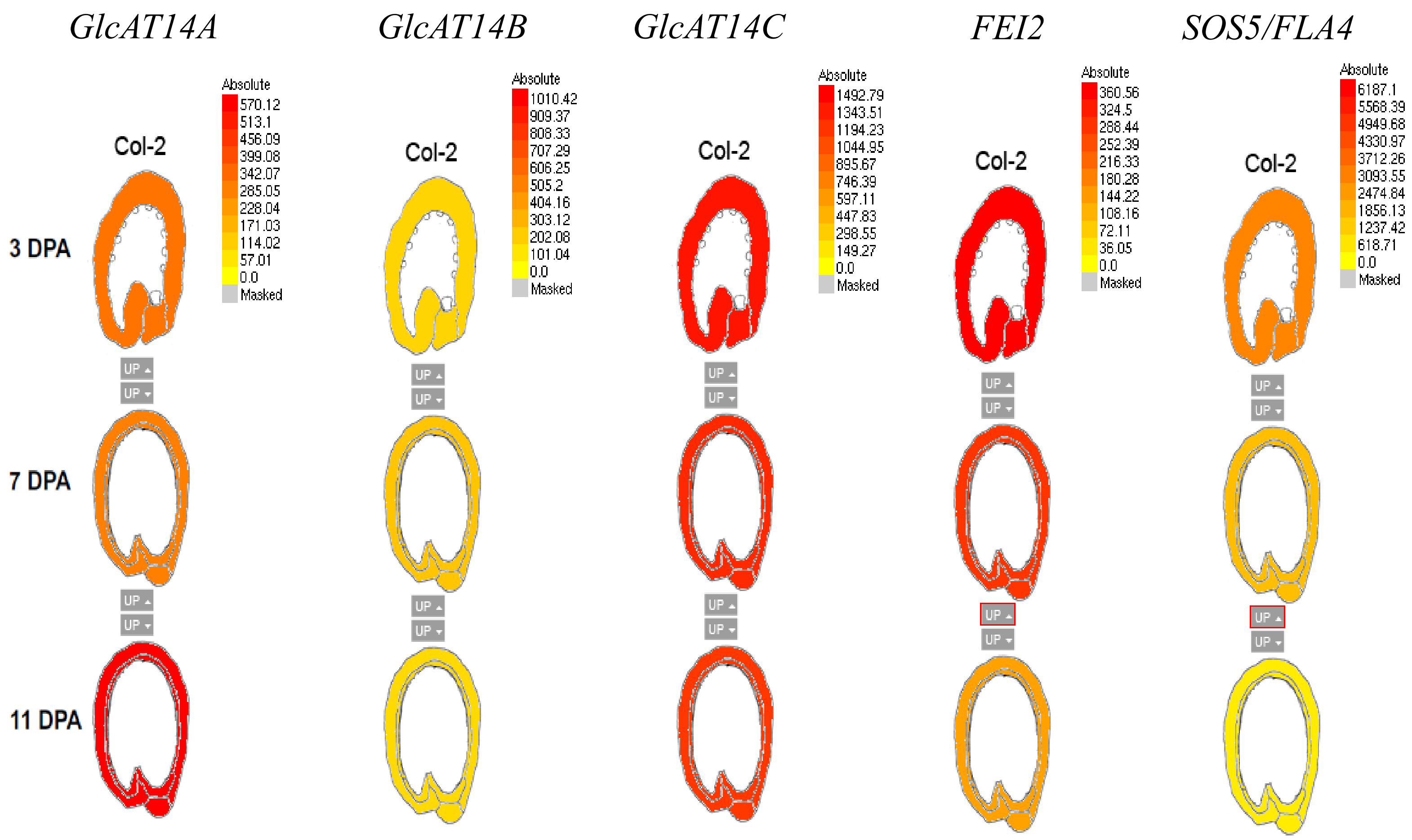

Supplementary Figure 2

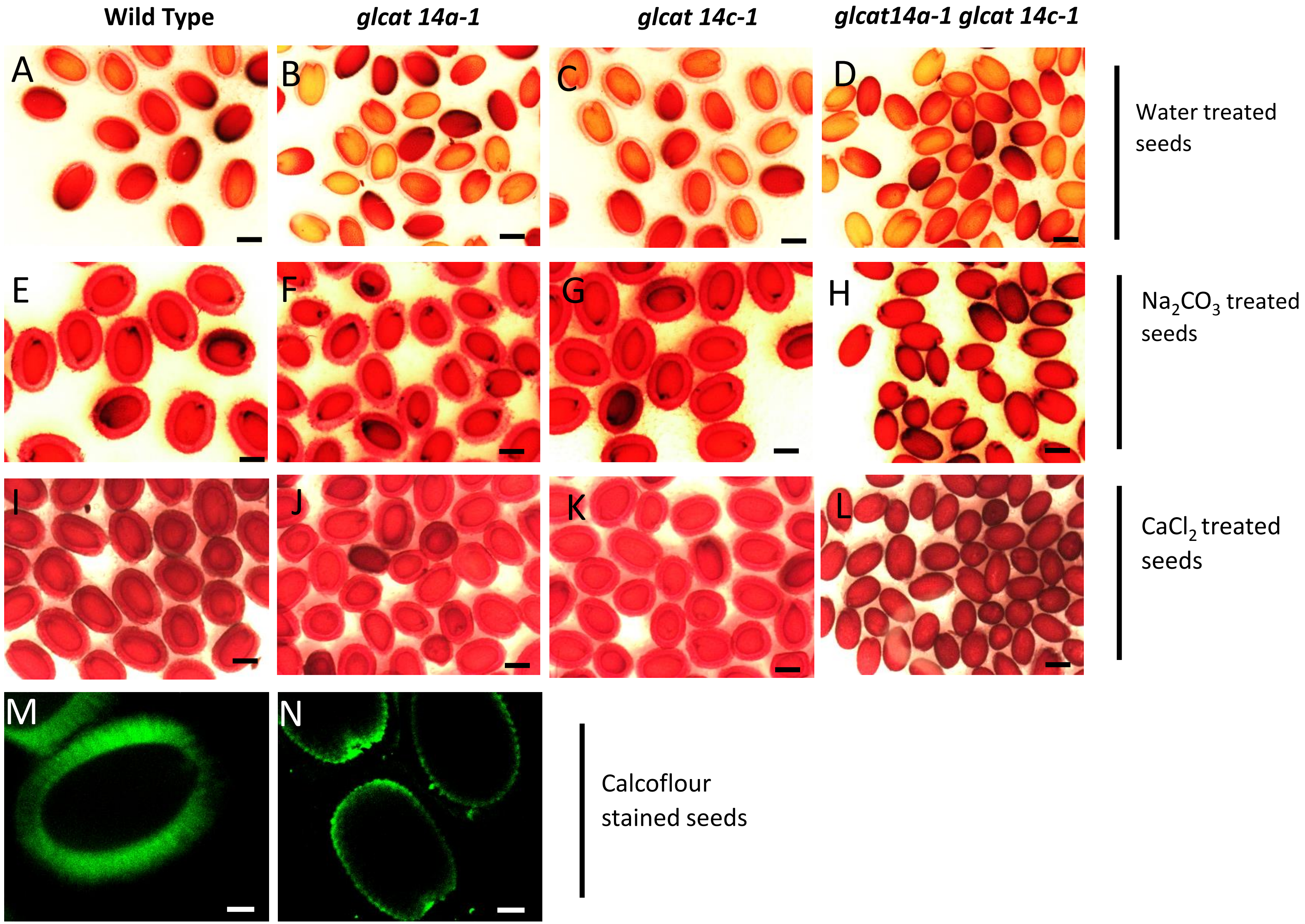

Supplementary Figure 3

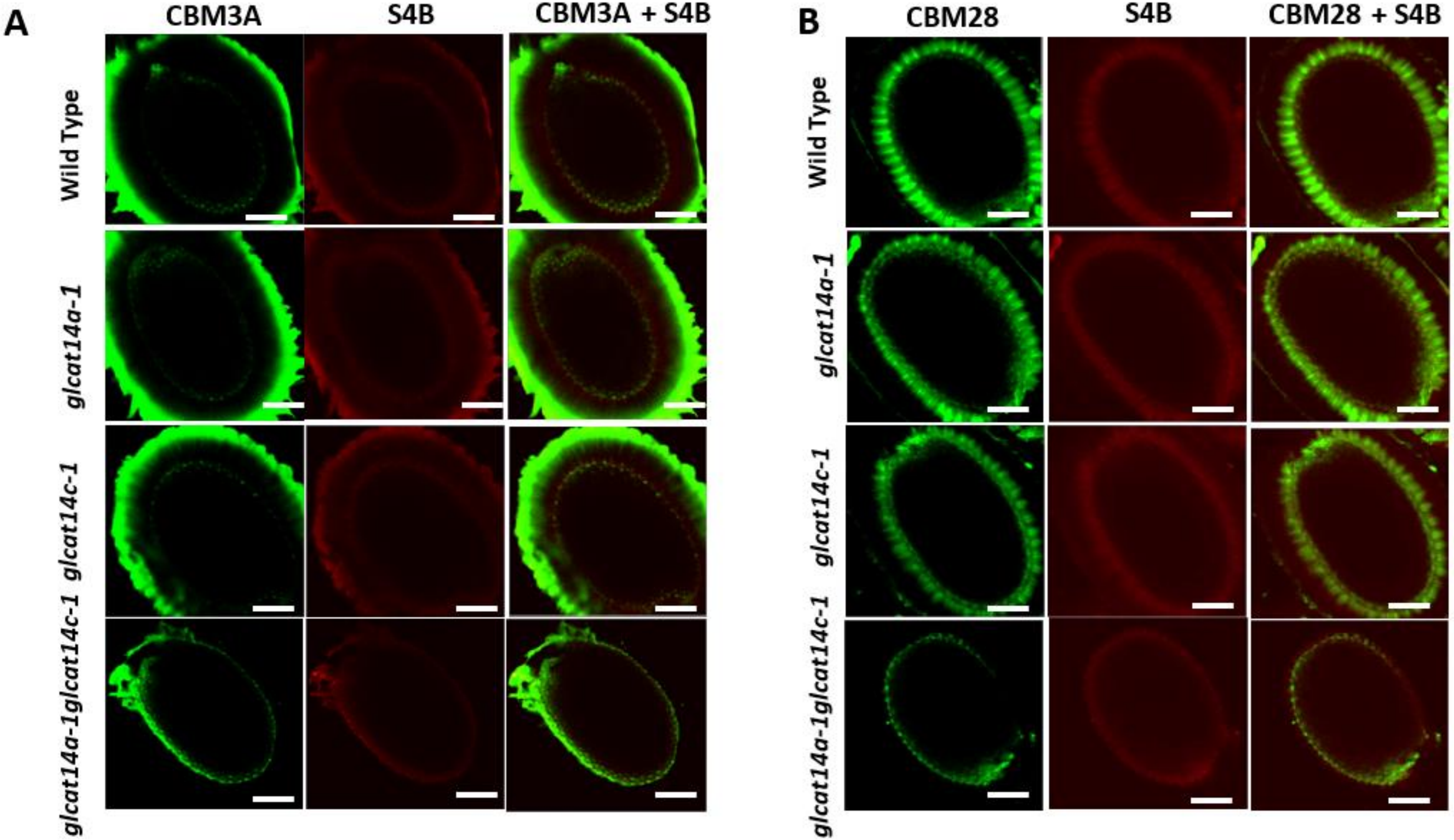

Supplementary Figure 4

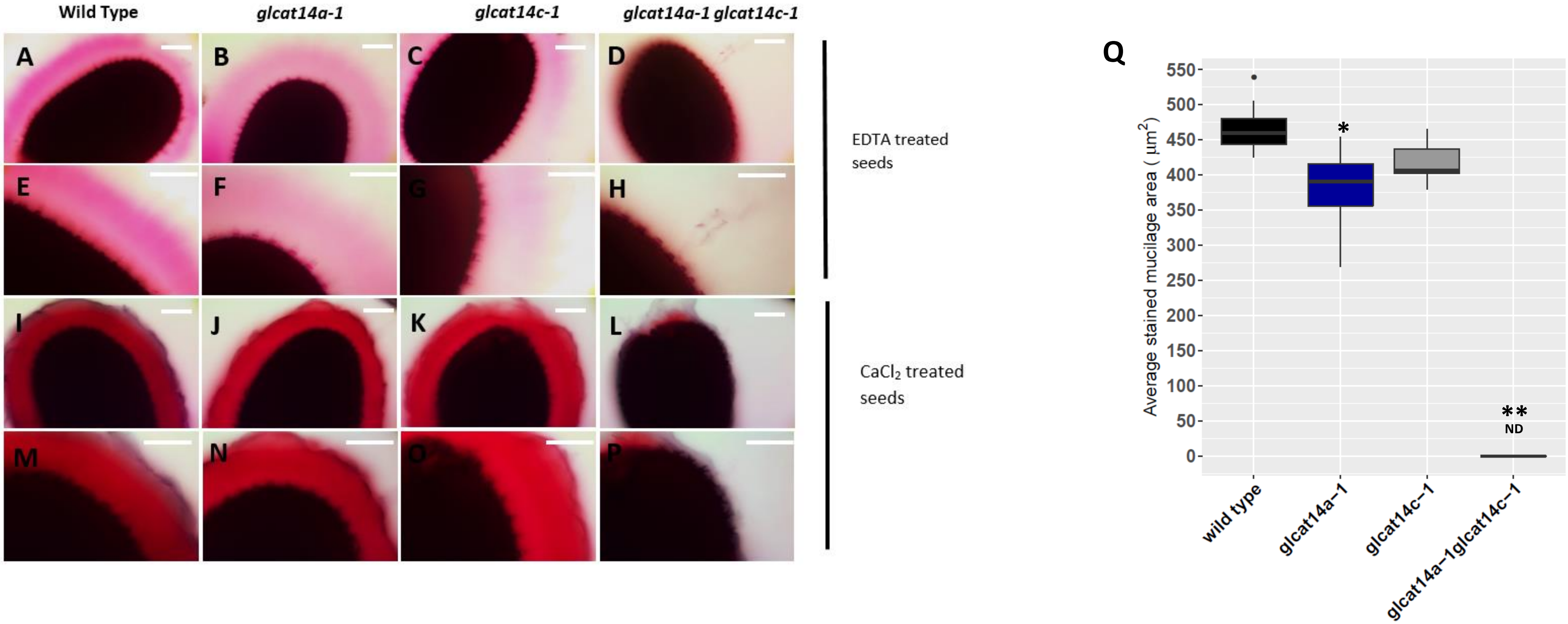

Supplementary Figure 5

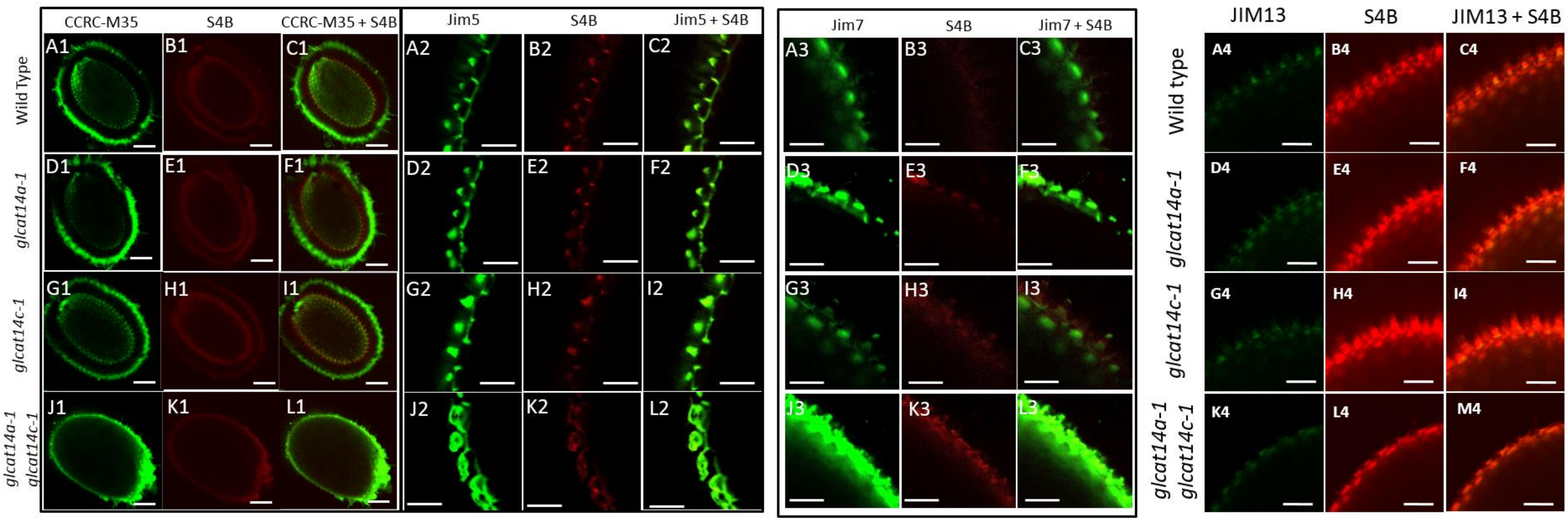

Supplementary Figure 6

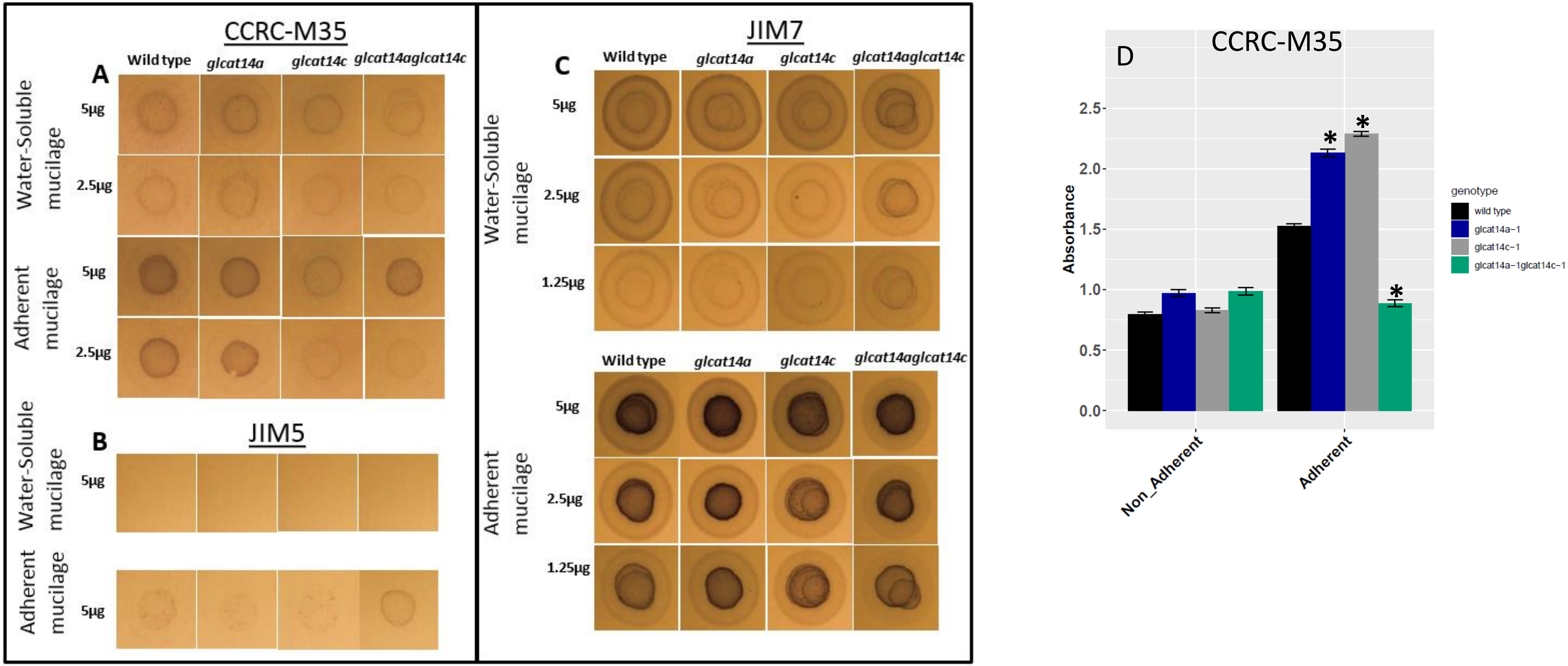

Supplementary Figure 7

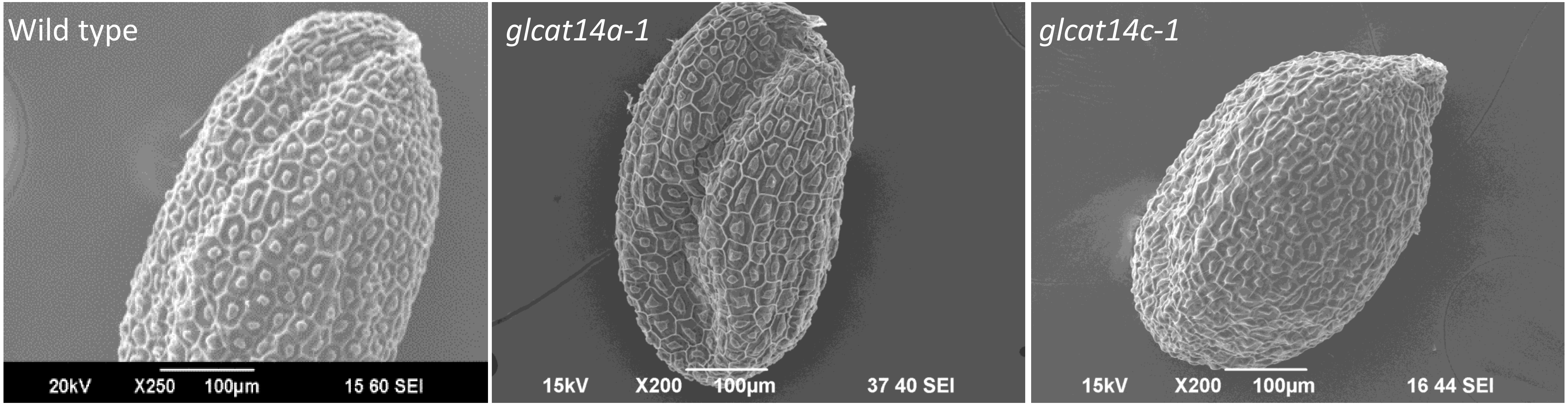

Supplement: Supplementary file 1 — Additional file 1: Supplemental Table 1. List of primers used for mutant characterization. Supplemental Figure 1. Expression of the GLCAT14A, GLCAT14B, GLCAT14C, FEI2 and SOS5 genes in the Arabidopsis seed coat. The Arabidopsis eFP browser (http://bar.utoronto.ca/efp_seedcoat/cgi-bin/efpWeb.cgi) was used to examine the expression of the GLCAT genes involved in glucuronidation of Type II AG and was compared to the expression of the FEI2 and SOS5/FLA4 genes in the seed coat at 3 day post anthesis (DPA), 7 DPA and 11 DPA. Supplementary Figure 2. Mucilage phenotypes of WT and glcat14 mutants hydrated in different chemical extractants. A-D, Staining of the adherent mucilage with 0.01% ruthenium red (RR) after vortexing briefly for 5 min in water (A-D), Na2CO3 (E-H) and 50mM CaCl2 (I-L).WT (M) and glcat14a-1glcat14c-1 (N) seeds were shaken in water and stained with calcofluor, which primarily stains cellulose, but also stains pectic galactan, xylan, and galactomannan to a lesser extent [26]. Images (A-L) were acquired using a light microscope, while images M and N were acquired using a Zeiss confocal microscope using the same acquisition settings to acquire both images. Three independent experiments (each with more than 25 seeds) were performed with similar results. Bar = 200μm for (A-L); Bar = 100 μm (M and N). Supplemental Figure 3. Immunolabeling of crystalline and amorphous cellulose in WT and glcat14 mucilage. Immunolabeling of crystalline cellulose with the CBM3a antibody (A) which binds preferentially to crystalline cellulose and the CBM28 antibody (B) which binds preferentially to amorphous cellulose in the adherent mucilage of the WT and glcat14 mutants [27]. The cellulosic ray-structure was counterstained with the S4B dye (red fluorescence). Three independent experiments (each with more than 25 seeds) were performed and similar results were obtained in each case. All scale bars = 50 μm. Supplementary Figure 4. Mucilage phenotypes of WT and glcat14 mutants. A-D, [file 12870_2021_3012_MOESM1_ESM.zip › Supp file_BMC Plant biology_reviewed.pdf]
